# Trends and determinants of newborn mortality in Kyrgyzstan: a Countdown country case study

**DOI:** 10.1016/S2214-109X(20)30460-5

**Published:** 2020-12-10

**Authors:** Mahdis Kamali, James E Wright, Nadia Akseer, Hana Tasic, Kaitlin Conway, Saman Brar, Cholpon Imanalieva, Gerrit Maritz, Arjumand Rizvi, Baktyiar Stanbekov, Sagynbu Abduvalieva, Elvira Toialieva, Zulfiqar A Bhutta

**Affiliations:** a Centre for Global Child Health, Hospital for Sick Children, Toronto, Canada; b UNICEF Kyrgyzstan, Bishkek, Kyrgyzstan; c Center of Excellence in Women and Child Health, Aga Khan University, Karachi, Pakistan; d Electronic Health Center, Ministry of Health, Bishkek, Kyrgyzstan; e National Center of Mother and Child Health, Ministry of Health, Bishkek, Kyrgyzstan; f Bishkek, Kyrgyzstan

## Abstract

**Background:**

Kyrgyzstan has made considerable progress in reducing child mortality compared with other countries in the region, despite a comparatively low economic standing. However, maternal mortality is still high. Given the availability of an established birth registration system, we aimed to comprehensively assess the trends and determinants of reproductive, maternal, newborn, and child health in Kyrgyzstan.

**Methods:**

For this Countdown to 2030 country case study, we used publicly available data repositories and the national birth registry of Kyrgyzstan to examine trends and inequalities of reproductive, maternal, and newborn health and mortality between 1990 and 2018, at a national and subnational level. Coverage of newborn and maternal health interventions was assessed and disaggregated by equity dimensions. We did Oaxaca-Blinder decomposition to determine the contextual factors associated with the observed decline in newborn mortality rates. We also undertook a comprehensive review of national policies and programmes, as well as a prospective Lives Saved Tool analysis, to highlight interventions that have the potential to avert the most maternal, neonatal, and child deaths.

**Findings:**

Over the past two decades, Kyrgyzstan reduced newborn mortality rates by 46% and mortality rates of children younger than 5 years by 69%, whereas maternal mortality rates were reduced by 7% and stillbirth rates by 29%. The leading causes of neonatal deaths were prematurity and asphyxia or hypoxia, and preterm small-for-gestational-age infants were more than 80 times more likely to die in their first month of life compared with those born appropriate-for-gestational age at term. Except for contraceptive use, coverage of essential interventions has increased and is generally high, with limited sociodemographic inequities. With scale-up of a few essential neonatal and maternal interventions, 39% of neonatal deaths, 11% of stillbirths, and 19% of maternal deaths could be prevented by 2030.

**Interpretation:**

Kyrgyzstan has reduced newborn mortality rates considerably, with the potential for further reduction. To achieve and exceed the Sustainable Development Goal 3 targets for newborn survival and reducing stillbirths, Kyrgyzstan needs to scale up packages of interventions for the care of small and sick babies, assure quality of care in all health-care facilities with regionalised perinatal care, and create a linked national registry for mothers and neonates with rapid feedback and accountability.

**Funding:**

US Fund for UNICEF under the Countdown to 2015, UNICEF Kyrgyzstan Office.

## Introduction

Kyrgyzstan is a lower-middle-income country of 6·2 million people, with over 100 ethnicities living in largely mountainous, rural terrains spanning eight provinces (*oblasts*).^1^, ^2^ After the break-up of the Soviet Union in 1991, Kyrgyzstan and several other central Asian republics were plunged into major economic crises.^3^ After a period of steep economic decline, the country transitioned to a market economy, although poverty rates still hover at about 25% of the population (appendix 6 p 1).^1^

Despite health-care reforms, Kyrgyzstan has one of the highest maternal mortality rates in the central Asian region, at 76 deaths per 100 000 livebirths in 2015.^4^ However, the mortality rate for children younger than 5 years has declined notably from 65 deaths per 1000 livebirths in 1990 to 21 in 2015, making Kyrgyzstan one of only 24 lower-middle-income countries to have achieved Millennium Development Goal (MDG) 4.^5^, ^6^

Kyrgyzstan is unique among lower-middle-income countries as it invested in a comprehensive civil registration and vital statistics system over a decade ago.^7^, ^8^ Piloted in 2009, Kyrgyzstan's Ministry of Health implemented systematic monitoring of newborn registration in select primary health-care centres, which was gradually rolled out to centres across the country by 2014. However, these data have not been used to inform policies to improve perinatal and newborn care. Registration of births and deaths is fundamental to social inclusion and human rights and to monitor progress of the Sustainable Development Goals (SDGs), particularly since 15 of 17 SDGs include indicators requiring civil registration and vital statistics data.^9^

**Research in context**

**Evidence before this study**

We did a systematic review to examine all published and unpublished (grey) literature for studies and data pertaining to reproductive, maternal, newborn, and child health interventions, and nutrition and mortality outcomes and determinants in Kyrgyzstan between 1990 and March 21, 2019. We devised and undertook a systematic strategy for implementation of the review. We searched relevant archives including MEDLINE, Embase, Scopus, Web of Science, and PubMed, for literature published from Jan 1, 1990, to March 21, 2019, with no language restrictions, using the following search strings: “(nutr* OR health OR mortality* OR mortalities OR death* OR fatal* OR survi* OR stillbirth* OR stillborn* OR diarrhea* OR pneumonia)”, “(child* OR infan* OR newborn* OR neonatal* OR neonate* OR matern* OR mother*)”, and “(Kyrgyz Republic OR Kyrgyzstan OR Kirghiz OR Kirghiz Soviet Socialist Republic OR Kirghizia OR Kyrgyz*)”. Two team members (MK and JEW) independently screened all studies at the title and abstract (656 studies) and full-text (45 studies) stages, with data extraction done in Excel. We did a grey literature search to find any remaining policies or programmes related to neonatal mortality. Websites or databases searched included Countdown, UNICEF, World Bank, WHO Library Database, WHO, UN Population Fund, UN Development Programme, Asian Development Bank, US Agency for International Development, GIZ, KFW, Swiss Agency for Development and Cooperation, UK Department for International Development, Swedish International Development Cooperation Agency, and the Ministry of Health of Kyrgyzstan. References and bibliographies of identified reports were also evaluated, as were relevant reports from development agencies and bilateral donors (appendix 6 pp 33–35). Altogether, two major national stakeholder consultations were held to share findings and obtain feedback. These data were triangulated and synthesised to develop a coherent and comprehensive overview of Kyrgyzstan's effective governance, role of development partners, and donor and other stakeholder initiatives that related to newborn health and survival gains in the country. We found that existing literature on reproductive health and on health, nutrition, and survival of mothers, neonates, and children in Kyrgyzstan was generally insufficient. Of the 45 published peer-reviewed articles that passed title and abstract screening, only 12 met the inclusion criteria, of which none comprised of a comprehensive review of reproductive, maternal, newborn, and child health in the country. Four studies attempted to review the health systems and policies in central Asia but did not highlight Kyrgyzstan nor did they assess the effect of such systems and policies on tangible outcomes. Three studies examined the drivers, equity of interventions, and causes of death of mothers, neonates, and children but all were global, multicountry assessments with no clear focus on Kyrgyzstan. The review showed that Kyrgyzstan had a steep economic and health decline after the collapse of the Soviet Union. Now, it is an exemplar Countdown country, achieving its Millennium Development Goal 4 target and having low levels of inequity in maternal, newborn, and child health interventions. However, the country is well documented to still struggle with the health-care delivery system, including insufficient qualified medical personnel and supplies, deficient facilities, and inadequate financial resources.

**Added value of this study**

To our knowledge, this was the first systematic and comprehensive analysis of reproductive, maternal, and newborn health in Kyrgyzstan. Our retrospective longitudinal assessment was powered by the use of national household health and demographic surveys, as well as primary datasets from the Kyrgyzstan's Ministry of Health national birth registry. The national dataset provided 5 years of complete civil registration and vital statistics across the country, a rare asset in low-income and middle-income countries. This unique analysis underscores the progress the country has made to reduce newborn mortality rates between 1990 and 2018, despite its changing social and economic circumstances. Progress is attributable to many factors, including high coverage of essential maternal and newborn health interventions, increased proportion of early initiation of breastfeeding, decreases in the number of children a woman has, and health sector reforms.

**Implications of all the available evidence**

Findings from this study could be used by policy makers, government officials, and development partners for accelerated action towards ending preventable maternal, stillborn, and newborn deaths by the end of the Sustainable Development Goals (SDGs) in 2030. Kyrgyzstan should build on its successes of reducing child mortality (SDG 3.2) to address ongoing health system challenges. To achieve all targets of SDG 3, Kyrgyzstan needs to strengthen its referral processes and prioritise assurance of quality of care for every neonate in all facilities across the country, in addition to scaling up packages of interventions for care of small and sick neonates (SDG 3.4). The country should ensure that essential reproductive, maternal, newborn, and child health services are available and accessible to all citizens (SDG 3.8) while also focusing on the improvement of maternal health, preconception care, and nutrition through improved antenatal care to avert many preventable deaths (SDG 3.1).

In this study, we aimed to investigate the trends and determinants of reproductive, maternal, newborn, and child health in Kyrgyzstan between 1990 and 2018, using birth registry and available survey data, and we considered implications of our findings for policy.

## Methods

### Study design

For this Countdown to 2030 country case study, we assessed overall and *oblast*-level trends in mortality rates of mothers, neonates, and children younger than 5 years; causes of death; and coverage of and expenditure on reproductive, maternal, newborn, and child health interventions. Stillbirths were defined as a birth with no signs of life after 20 weeks of gestation, while neonates were defined as babies aged 0–28 days. We also modelled the number of lives that could be saved with a scale-up of these interventions. Ethics approval was received from the Hospital for Sick Children (Toronto, Canada), and a data transfer agreement was entered into with the Ministry of Health of Kyrgyzstan for the use of anonymised data for our study.

### Data sources and management

We reviewed all online data repositories with good quality information on reproductive, maternal, newborn, and child health and determinants in Kyrgyzstan (appendix 6 p 1). We obtained national estimates of child mortality and causes of death from the UN Inter-Agency Group for Child Mortality Estimation (UN-IGME),^5^ the Institute for Health Metrics and Evaluation (IHME) for 1990–2018,^10^ and the UN Maternal Mortality Estimation Inter-agency Group for 1990–2015.^4^ We analysed household-level data from Multiple Indicator Cluster Surveys (MICS)^11^, ^12^, ^13^ from 2006–18 and Demographic and Health Surveys (DHS)^14^, ^15^ from 1997–2012 for relevant outcomes.

Through an agreement with the Ministry of Health of Kyrgyzstan, we obtained the anonymised national birth registry (NBR) data for 2010–17. Under a national regulation, this birth registry collects information relating to maternal and newborn health, birthweight, and gestation outcomes, and pregnancy, stillbirths, and newborn complications and causes of death for all livebirths heavier than 500 g from all facilities in the country. Mortality information was also provided for 920 neonates aged 30 days to 1 year who had died outside of health facilities in 2013–17 from an additional infant mortality registry of the Ministry of Health.

We found that the number of births in the NBR in 2013–17 were comparable with the births estimated by the UN Population Division (difference range 0–3%;^16^ appendix 6 p 2), and the overall subnational distribution (appendix 6 p 3), with the exception of Jalal-Abad, where implementation was not complete until 2014. Excluding the pilot phase of birth registration, we used the data from 2013–17 to inform newborn and child mortality rates, stillbirths, and causes of death. Additionally, we assessed coverage and inequalities of key maternal and neonatal health interventions using standard Countdown methods and definitions^17^ (appendix 6 pp 3–4). Data sources were all weighted equally and compared for various outcomes.

### Statistical analysis

Individual-level age-specific mortality counts and total livebirth estimates from the NBR were derived and compared with the global and Kyrgyzstan-specific estimates from UN-IGME,^5^ MICS,^12^, ^13^ and DHS.^14^, ^15^ We disaggregated neonatal mortality rates by geographical area (*oblast* and district [*rayon*] level), area of residence (rural *vs* urban), and wealth (asset indices). For ease of interpretation, wealth quintile data were collapsed for the bottom two quintiles and top two quintiles. We assessed coverage of core maternal and neonatal health interventions along the continuum of care using data collected from MICS 2018,^13^ and for trend analysis compared with available estimates from DHS 2012 and DHS 1997.^14^, ^15^

Leading causes of death for neonates and stillbirths estimated from the NBR for the years 2013–17 were combined and compared with modelled estimates from UN-IGME^5^ and the Child Health Epidemiology Reference Group (CHERG).^18^ Codes from the International Classification of Diseases, tenth edition used in the NBR were combined into seven groups (asphyxia, congenital malformations, hypoxia, infection, prematurity, respiratory, and other) with the same categorisations used by UN-IGME.^5^

Given known associations with outcomes,^19^ we assessed the risk of various birth outcomes by stratifying estimates of newborn mortality rates in four categories by use of generalised linear modelling with a log-link function: neonates born at full term (≥37 weeks of gestation) and small for gestational age, preterm (<37 weeks) and at an appropriate weight for their gestational age, preterm and small for gestational age, and full term and at an appropriate weight for their gestational age. The weight-for-gestational-age classification for each neonate was calculated with the INTERGROWTH-21st Newborn Size calculator.^20^

We did an Oaxaca-Blinder decomposition^21^ to determine the sociodemographic and contextual factors associated with the observed decline in newborn mortality rates using household-level information from DHS 1997 and MICS 2018, with a focus on births and newborn deaths over the 5 years preceding each survey.

We developed a conceptual framework regarding hierarchical determinants of newborn survival^22^, ^23^, ^24^, ^25^ (appendix 6 p 4), and we used multilevel, mixed-effects logistic regression in combination with hierarchical model building strategies, with newborn mortality rates as the dependent variable. Initially, unadjusted associations between each of the independent variables and newborn mortality rates were calculated, retaining any variable with p<0·20. Variables with an adjusted p<0·15 were further retained and adjusted for multi-collinearity by use of variance inflation factors, with more than three factors considered indicative of high intervariable correlation. Coefficients from this final model were multiplied by the change in proportions for each sociodemographic factor between 1997 and 2018, summed, and exponentiated to obtain relative risk (RR) estimates at each level individually and overall.^26^ All analyses were done with STATA, version 14.0, and were adjusted for survey design and sampling weights.

### Assessment of policies and interventions

We reviewed all original policies, laws, legislation, and strategies and programme documents pertaining to newborn health, MDG progress, and SDG planning in Kyrgyzstan, and we assessed their relationship to seven essential newborn intervention packages: preconception nutrition care, antenatal care, advanced antenatal care, care during labour and childbirth, immediate newborn care, care for healthy neonates, and care for small and sick neonates. We assessed national expenditure on reproductive, maternal, newborn, and child health using official development assistance data from the Countdown database and additional information on domestic financing obtained from the World Bank.^1^, ^27^ Two major national stakeholder consultations were held to share findings and obtain feedback.

We assessed the effect of systematically increasing coverage of various evidence-based interventions on reducing the burden of maternal, fetal, newborn, and child deaths using the Lives Saved Tool (LiST),^28^ which uses effectiveness estimates of interventions outlined in several *Lancet* Series.^29^, ^30^, ^31^, ^32^ Baseline coverage of interventions were taken from MICS 2018,^13^ and where estimates were not available, LiST default coverage estimates for Kyrgyzstan were used. We modelled scale-up of coverage from most recent figures to 90% in 2019–25, enhanced to 99% in 2026–30, given the national commitment of Kyrgyzstan to universal health coverage.

### Role of the funding source

The funders of the study had no role in study design, data collection, data analysis, data interpretation, or writing of the report. All authors had full access to all the data in the study. The corresponding author had final responsibility for the decision to submit for publication.

## Results

**Figure 1:**
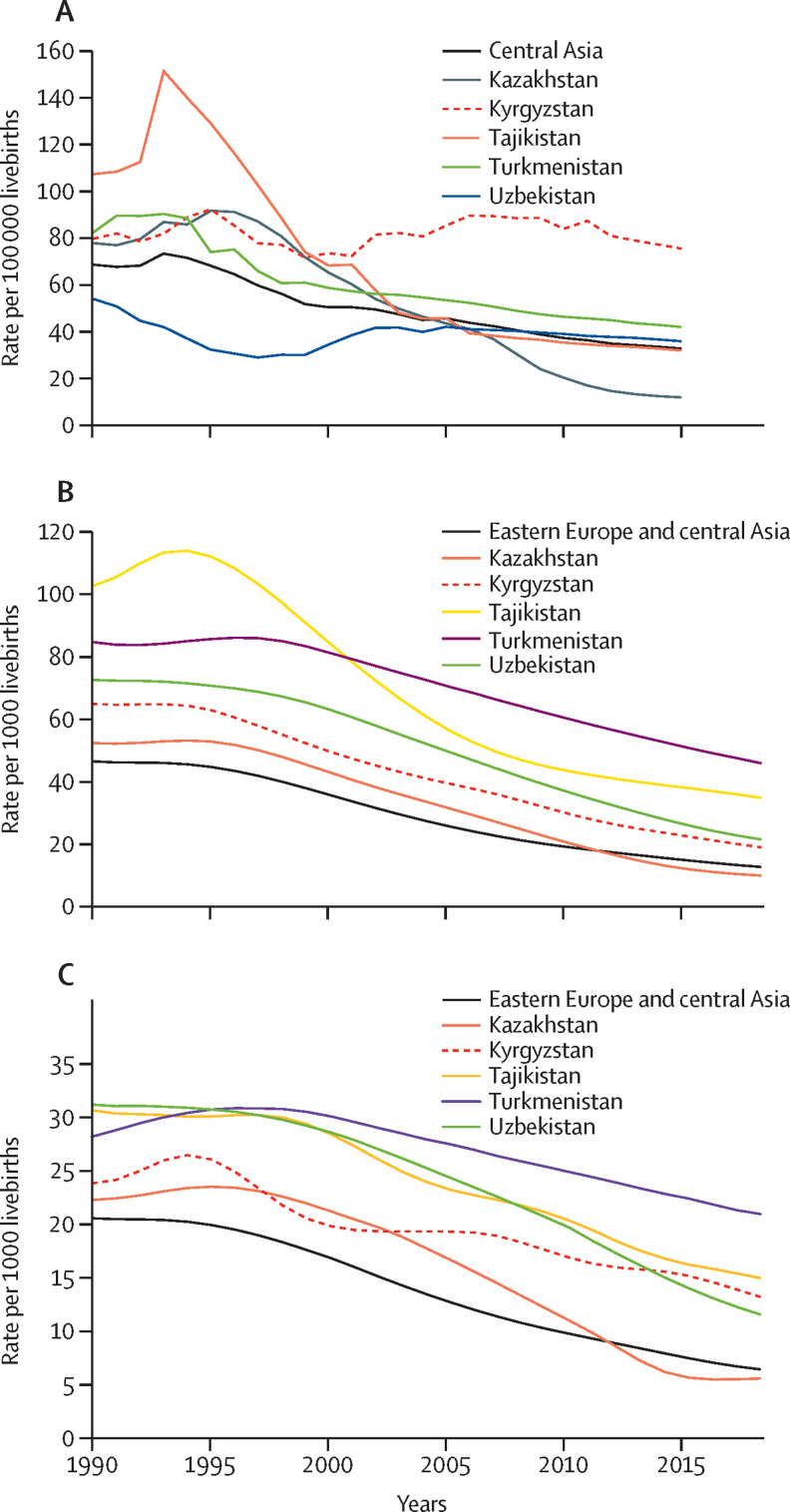
Trends in mortality in Kyrgyzstan and the central Asia region, for mothers (A), children younger than 5 years (B), and neonates (C)

During the MDG period (1990–2015) in Kyrgyzstan, maternal mortality was reduced by only 7% (from a rate of 82 to 76 deaths per 100 000 livebirths) compared with 54% for the central Asian region (from 69 to 32 per 100 000; figure 1A, appendix 6 p 5). Stillbirth rates in Kyrgyzstan remained fairly constant, decreasing from about 14 per 1000 births in 2000, to 10 per 1000 by 2015 (appendix 6 p 5); mortality rates of children under 5 declined by 69% between 1990 and 2017, from 64·8 to 20·0 per 1000 livebirths (figure 1B; appendix 6 p 6); and newborn mortality rates declined by 46% (from 24 per 1000 livebirths in 1990 to 13 in 2017; figure 1C), compared with a 70% decline in the region (from 21 per 1000 livebirths in 1990 to 6 in 2017; appendix 6 p 6).

Newborn mortality rates varied between *oblasts* in the country (appendix 6 p 7). Consistently, between 2013 and 2017, Osh City had high newborn mortality rates (ranging from 35 to 26 per 1000 livebirths), whereas Chui, Osh, and Issyk Kul *oblasts* had rates lower than 10 per 1000 livebirths. These findings might reflect relative population density and birth rates. The largest overall reductions in newborn mortality rates at *oblast* level during 2013–17 were observed in Osh City, followed by Chui, Batken, and Talas, whereas the smallest improvements occurred in Bishkek (appendix 6 p 8). Given the restricted range of annual data from NBR, we used survey data to examine the *oblast*-level annual rate of change of newborn mortality rates over an 11-year period. Talas had the greatest improvements in newborn mortality, from a rate of 72·8 per 1000 livebirths in 1997 to 9·3 in 2018, whereas Naryn had the least reduction, with an annual rate of change of 0·3 per 1000 livebirths. Overall, inequalities between *oblasts*, and inequalities related to maternal education and wealth groups have also reduced since 1997 (appendix 6 pp 9–10).

**Figure 2:**
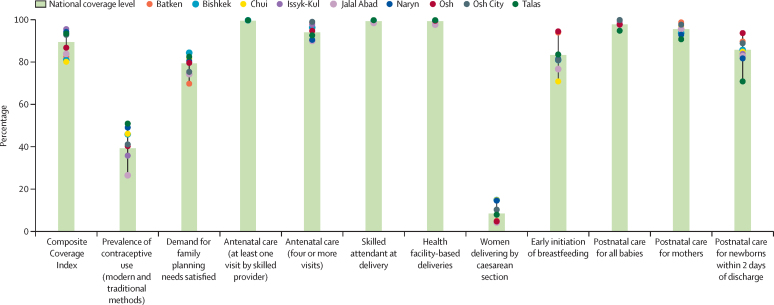
Coverage of neonatal and maternal interventions by province (oblast), MICS 2018 MICS=Multiple Indicator Cluster Surveys.

Coverage of most essential maternal and newborn health interventions was high in 2018 (figure 2), but subnational variations existed, and prevalence of contraceptive use was estimated to be lower than 40% nationally, ranging from 51% in Talas to just 26% in Jalal Abad. Other than modern contraceptive use and postnatal care for neonates and mothers, inequalities in access to interventions were negligible by wealth or residence (appendix 6 pp 11–12). Coverage of interventions have increased over time and inequalities with respect to wealth and rurality decreased, except for prevalence of contraceptive use, which decreased from 59% nationally in 1997, to 39% in 2018; and postnatal checks, which decreased from 64% in 2014, to 49% in 2018. Notwithstanding its importance, we found very little information available on quality of care across interventions (appendix 6 p 13).^33^, ^34^

All sources (CHERG, IHME, and NBR) showed prematurity as the leading cause of neonatal deaths (41–47% of deaths; appendix 6 p 15). The second biggest cause of neonatal death was asphyxia or hypoxia; however, the proportions varied across the sources (15·0% in the NBR compared with 25·0% in CHERG and 32·8% in IHME). Another difference between the sources were the proportion of deaths caused by infections (12·8% in the NBR compared with 7·7% in CHERG and 2·8% in IHME). The registry allowed for greater detail regarding cause of death than modelled data. Between 2013 and 2017, the leading causes of neonatal death among normal birthweight infants (≥2500 g) were congenital malformations (26·5%), followed by asphyxia or hypoxia (22·8%) and infections (18·2%), while for low birthweight infants (<2500 g), leading causes were prematurity (55·3%), and asphyxia or hypoxia (13·8%; appendix 6 p 18). The category of other causes included several instances of trauma and injury at birth and intraventricular haemorrhage (appendix 6 pp 16–17).

40·7% of all neonatal deaths in Kyrgyzstan between 2013 and 2017 occurred within the first 24 h of life, with over two thirds (69·1%) in the first 2 days (appendix 6 p 18). 85·1% of stillbirths between 2013 and 2017 were classified as occurring during the antenatal period (appendix 6 p 18). Full-term neonates (≥37 weeks) who were small for gestational age had over 4 times the risk of dying in the first 28 days of life (RR 4·38, 95% CI 3·98–4·81) compared with those who were full-term and appropriate for their gestational age (appendix 6 p 19). The greatest risks of neonatal death were observed in preterm (<37 weeks) neonates appropriate for their gestational age who, compared with full-term infants appropriate for their gestational age, were almost 60 times more likely to die in the first month of life (RR 57·5, 95% CI 54·8–60·5); and in preterm small-for-gestational-age babies, with over 80 times the risk of death in the first 28 days of life (83·2, 77·1–89·8).

The decomposition analysis showed that 42**·**9% of the change in neonatal mortality rates could be attributed to the increase between 1997 and 2018 in the proportion of infants being fed by breastfeeding within 1 h of birth, closely followed by the overall reduction in maternal parity (30·3%; appendix 6 pp 20–21). Collectively, the sociodemographic and contextual variables retained in the final model explain 83% of the observed decrease in neonatal mortality rates.

Despite Kyrgyzstan's early investments in primary care in the Soviet period, post-independence policies related to reproductive, maternal, and newborn health were largely ignored until 2006, when primary care reform, *Manaas Taalimi*, was implemented (appendix 6 pp 22–30). The National Reproductive Health Strategy was launched in the same year, and the National Perinatal Health Programme soon thereafter, in 2008. The majority of programmes and policies referred to the seven essential newborn intervention packages broadly and did not stipulate specific interventions. The national health reform programme *Den Sooluk*, and the national perinatal care programme included all seven essential newborn intervention packages and stated explicitly the interventions related to each. Other programmes specified interventions related to one package, with five touching on breastfeeding initiatives, two relating to the preconception nutrition care package, and only one programme focusing on the care of small and sick neonates. Overall, few programmes addressed interventions with a direct effect on the major contributors to neonatal mortality. Kyrgyzstan's dependency on external aid is high; over 40% of the country's expenditure in 2015 was from official development assistance, although this dropped to about 25% in 2016 and 2017 (appendix 6 p 31). Kyrgyzstan has consistently spent much more on child health than on maternal and neonatal health.

**Figure 3:**
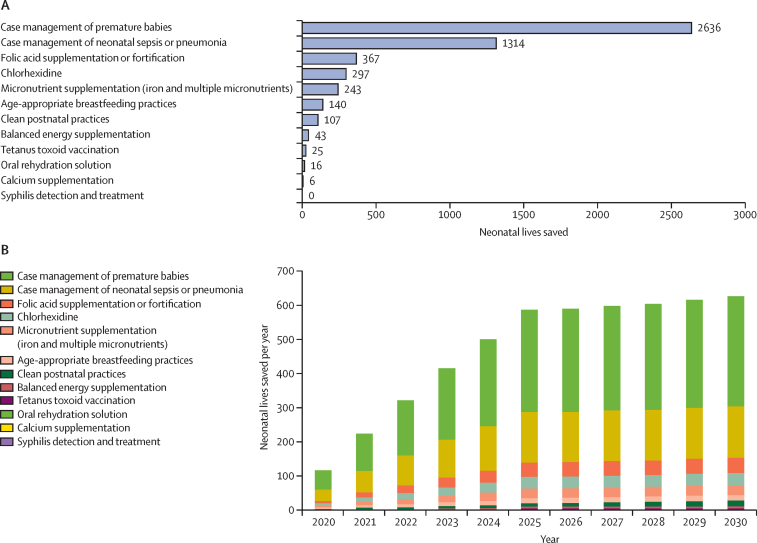
Estimated number of neonatal lives saved in 2020–30 assuming scale-up to 90% by 2025 and 99% by 2030 Total number of neonatal lives saved by 2020–30 nationally (A) and by type of intervention annually (B).

Projecting from the baseline data used for LiST, we showed that, with scale-up of Every Newborn Action Plan interventions,^31^ 38·9% of neonatal deaths could be prevented by 2030, along with 11·2% of stillbirths and 18·6% of maternal deaths (appendix 6 p 32–34). Improved case management of premature babies could have the greatest effect on the number of neonatal deaths over the decade, saving more than 2500 neonates (figure 3A). By 2025, this intervention might save an average 300 neonatal lives per year (figure 3B), while increased access to micronutrient supplementation might save the most maternal lives and prevent the most stillbirths (appendix 6 p 35).

## Discussion

Our analysis suggests that, despite the post-independence economic downturn, Kyrgyzstan has reduced neonatal mortality rates by 46% since 1990. However, the decline in these rates was variable across the country and, with additional targeting and improvements in quality of care for childbirth, neonates, and postnatal care in rural *rayons*, much more could be achieved. Given the insights from the NBR into prematurity and respiratory distress as major causes of neonatal deaths, investments in quality of care for mothers and neonates, appropriate referral systems, and facility-based care could help improve perinatal outcomes.

We should recognise several possible data limitations in our study. While the overall annual births captured in the NBR and neonatal mortality rate estimates compared well with the UN Population Division and UN-IGME figures, some of the categories of causes of death could be inaccurate. Given the potential for disciplinary action, a legacy of the Soviet era, systematic under-reporting of intrapartum stillbirths might occur. The timing of death for stillbirths has not changed much over the years, with more than 80% of stillbirths occurring in the antenatal period. Additionally, relatively few deaths were related to neonatal infections or sepsis in the NBR, which could relate to similar under-reporting of neonatal sepsis as a cause of neonatal mortality. This low estimate could also relate to the scarcity of sophisticated microbiological facilities and standardised protocols for recognition and management of neonatal sepsis. Osh City and Bishkek had the highest neonatal mortality rates, potentially due to referrals of high-risk pregnancies to the only tertiary-care facilities in these *oblasts*, although such transfers from other facilities or regions were not clearly documented in the NBR.

Although we attempted to link maternal health and morbidities in the available household surveys and NBR, information regarding the contributing maternal conditions to birth and neonatal outcomes was inadequate and non-standardised. The maternal mortality rate in Kyrgyzstan is exceptionally high, possibly owing to the completeness of vital registration systems compared with those in other countries in the region. This issue warrants additional research. Our analysis supports the need to improve linkages between maternal health, quality of care, and neonatal survival. The high proportion of deaths and complications among neonates born preterm and small for their gestational age is probably explained by the mother's health and factors related to lifestyle, nutrition, or environmental exposures.^35^, ^36^, ^37^, ^38^ The NBR data can be substantively improved as a combined registration system for mothers and neonates, with the potential to capture an expanded repertoire of coverage indicators for women antenatally and postnatally, collecting quality data on maternal health, nutrition, and quality of care. A linked dataset would be beneficial for many reasons, including the identification of specific geographical areas for targeting and regionalisation of maternal and newborn health care, as well as for identifying causes of stillbirths.

Kyrgyzstan has implemented some important policies and programmes related to maternal and newborn health, including the *Manaas* (1996–2006) and *Manaas Taalimi* (2006–10), the National Reproductive Health Strategy (2006–15), *Den Sooluk* (2012–16), and the National Perinatal programmes (2008–18). Overall, Kyrgyzstan appears to have a range of programmes that touch on each of the seven essential newborn intervention packages, both directly and indirectly, and are essential components of universal health care for mothers and neonates, with the potential for consolidation.

Notwithstanding these programmes, quality of care remains an issue. The World Bank's multi-year project assessing the effects of results-based financing and enhanced supervision on maternal and newborn health service quality, evaluated all 65 secondary hospitals in Kyrgyzstan at baseline in 2012.^39^ Gaps in quality antenatal care included basic antenatal care supplies (eg, tetanus toxoid vaccines and iron or folic acid tablets), history-taking and health status assessments during visits, and birth counselling. However, reassuringly, tracer drug availability, infection control and sterilisation, supplies for routine labour and delivery and newborn care, and equipment for assisted deliveries were widely available in nearly all facilities.^39^ Therefore, Kyrgyzstan is well placed to augment solid primary care services and infrastructure with quality newborn care health services in each *oblast* to improve outcomes.

Several challenges persist in the reduction of neonatal mortality rates in Kyrgyzstan related to infrastructure, human resources, funding, collaboration, equity, and referral processes. The country's ageing perinatal infrastructure has been cited as a challenge in the provision of care to mothers and neonates.^40^, ^41^, ^42^, ^43^ The health-care system is constrained by antiquated infrastructure combined with insufficient funding for modernisation. Although the electronic newborn registry is now implemented across the country as a progressive health information system, recording and reporting forms were noted to be non-compliant with clinical protocols, and additional logbooks are kept by many hospitals, adding to the load of documentation.^44^ Our analyses have highlighted probable misclassification of timing of stillborn and neonatal death, as well as their causes, particularly for preterm infants. Sound and transparent maternal and perinatal audit systems would greatly enhance the utility of such data systems and quality control.

Human resources are another key bottleneck because of substantial staffing deficits.^42^, ^45^ There is a dearth of gynaecologists, obstetricians, and neonatal paediatricians in rural areas, with most concentrated in Bishkek and Osh.^42^ Reasons for poor staffing include low salaries, low incentives to work in rural areas, and outmigration.^41^, ^42^ Up-to-date neonatal training programmes for health professionals, especially nurses, physicians, and midwives, are needed. Collaborative linkages with leading academic centres across the region and globally could help to develop accredited fellowship training programmes in neonatal paediatrics and neonatal nursing.

Additional opportunities exist to reduce inequities at primary care level, through task sharing with community health workers. A systematic review assessing strategies to broaden the scope and reach of reproductive, maternal, newborn, and child health interventions in mountainous and hard-to-reach areas found that task shifting, strengthened roles of community health workers and mobile teams, and health promotion and awareness initiatives are very effective.^46^ The Village Health Committee And Community Action for Health Model in Kyrgyzstan is recognised as a key strategy for community mobilisation and health promotion, and could be very useful for addressing maternal and newborn health care in remote areas.^47^, ^48^

Although equitable financing for health care is highlighted in the National Health Reform Programme (*Den Sooluk*),^49^ inadequate health sector financing remains a challenge.^41^, ^45^, ^50^ Almost half of the country's total health expenditure represents out-of-pocket payments, thereby placing the financial burden of health care on patients and families.^51^ The country's dependency on external aid is high, as it constitutes a fourth of the country's total expenditure. Of the official development assistance received, only US$4 per capita is spent on maternal and newborn health, less than half of the amount spent on child health. A particular focus is needed on improving contraceptive use in the country, with fewer than 40% of women using contraception on average in Kyrgyzstan in 2018, a drop from 60% in 1997. The reasons for reduced coverage of modern contraception methods could also relate to dependence on external assistance for such commodities, and domestic financing needs to support such services.

A scoping review of health services delivery in Kyrgyzstan found that suboptimal organisation and coordination of services affected regular or timely access to care.^44^ Regionalisation of perinatal and newborn health services could help to develop centres of excellence for complicated maternal and newborn health care and training,^50^ but it might be more efficient for larger tertiary care hospitals across the country to operate as referral perinatal care centres, specialising in the care of small and sick newborn babies, coupled with sound transport systems at the *oblast* level with triage and transport of high-risk births to regional centres of excellence.

Kyrgyzstan is a remarkable example of a sound investment in a national birth registry and is well positioned to use this wealth of information in an effort to understand the barriers to newborn survival and the relevant targets of SDG 3 well before 2030. Moving forward, specific actions for addressing the residual burden of maternal and neonatal morbidity and mortality will yield the maximum benefit, requiring a national strategy specifically focused on scaling up packages of interventions for the care of small and sick babies, assuring quality of care, and regionalisation of maternal and newborn care.

## Data sharing

We were allowed access to the anonymised Kyrgyzstan birth registry for this specific analysis by the Ministry of Health, and we do not have authorisation to share it further. Interested researchers can apply to the Kyrgyzstan Ministry of Health for direct access to this birth registry and its updates.

## Supplementary Materials

For the Kyrgyz translation

For the Russian translation

For the French translation

For the Arabic translation

For the Spanish translation
