# Supplementary material for: Trends and determinants of newborn mortality in Kyrgyzstan: a Countdown country case study
Source: Lancet Glob Health. 2020 Dec 10;9(3):e352–60. doi: 10.1016/S2214-109X(20)30460-5 (PMC7886658; doi:10.1016/S2214-109X(20)30460-5)
Supplement: For the Kyrgyz translation [file mmc1.pdf]

THE LANCET
Global Health

Supplementary appendix 1

This translation in Kyrgyz was submitted by the authors and we reproduce it as supplied. It has not been peer reviewed. *The Lancet*'s editorial processes have only been applied to the original in English, which should serve as reference for this manuscript.

Бул кыргыз тилиндеги котормону авторлор камсыз кылышкан жана биз ал кандай формада сунуштасак, ошол формада жарыялаганбыз. Бул котормо эксперт тарабынан карала элек. Lancet редакциялык процесси англис тилиндеги түпнуска текстине карата гана жүргүзүлгөн, бул басылмага шилтеме катары каралышы керек.

Supplement to: Kamali M, Wright JE, Akseer N, et al. Trends and determinants of newborn mortality in Kyrgyzstan: a Countdown country case study. *Lancet Glob Health* 2020; published online Dec 10. http://dx.doi.org/10.1016/S2214-109X(20)30460-5.

Кыргыз Республикасындагы жаңы төрөлгөн ымыркайлардын өлүмгө учуроо тенденциялары жана көрсөткүчтөрү: Өлкөлүк тематикалык изилдөө үчүн кайтарым эсептөө

Махдис Камали[1], Джеймс Э. Райт[1], Надия Аксиир[1], Хана Тасик[1], Кэйтлин Конвэй[1], Саман Брар[1], Чолпон Иманалиева[2], Геррит Маритз[2], Арджуманд Ризви[3], Бахтияр Станбеков[4], Сагынбу Абдувалиева[5], Эльвира Тоялиева[6], Зульфикар А. Бхутта [1,3*]

[1] *Балдардын глобалдык ден-соолугу борбору, Оорукчан балдар үчүн госпиталь, Торонто, Канада*

[2] *ЮНИСЕФ, Кыргызстан*

[3] *Аялдар менен балдардын ден-соолугун коргоо боюнча бөлүм, Ага Хан университети, Карачи, Пакистан*

[4] *Электрондук Саламаттык сактоо Борбору, Саламаттык сактоо министрлиги, Кыргызстан*

[5] *Эне менен баланы коргоо улуттук борбору, Саламаттык сактоо министрлиги, Бишкек, Кыргызстан*

[6] *Перинаталдык кам көрүү боюнча көз карандысыз эксперт, Бишкек, Кыргызстан*

Аннотация

Өбөлгөлөр: Кыргыз Республикасы калктын жан башына болгон ИДП төмөн болгонуна карабастан, региондогу башка өлкөлөргө салыштырмалуу балдардын өлүмү көрсөткүчүн кыйла азайтууга жетишти. Төрөлүүнү каттоонун белгиленген тутуму болушунун аркасында биз жаңы төрөлгөн ымыркайлардын өлүмгө учуроо тенденциялары менен көрсөткүчтөрүн комплекстүү баалоодон өткөрдүк.

Ыкмалары: Биз улуттук жана субулуттук деңгээлде 1990-2018-жылдар аралыгында репродуктивдүү сааматтыктагы, эненин ден-соолугундагы жана жаңы төрөлгөн ымыркайлардын ден-соолугу менен өлүмгө учуроосундагы тенденциялар менен теңсиздиктерди изилдөө үчүн жеткиликтүү маалыматтар топтомун жана калктын

уникалдуу бирдиктүү мамлекеттик реестрин (төрөлүү реестри) пайдаландык. Эненин ден-соолугун жана жаңы төрөлгөн ымыркайлардын ден-соолугун коргоо боюнча кийлигишүүлөрдү камтуу теңчилик аспектилеринин алкагында бааланып, дезагрегацияланды. Оаксак-Блиндер динамикалык жайгаштыруу ыкмасы байкалып жаткан неонаталдык өлүмгө учуроо көрсөткүчүнө байланышкан контекстуалдык факторлорду аныктоо үчүн пайдаланылды. Биз ошондой эле көбүрөөк өмүрлөрдү сактап калууга дарамети бар кийлигишүүлөргө көңүл бурдуруу үчүн улуттук саясаттар менен программаларга комплекстүү баяндама жасап, сакталган өмүрлөрдүн санын баалоо үчүн шайманга (LiST) перспективалык талдоо жүргүздүк.

Жыйынтыктар: Акыркы эки декада ичинде негизги инвестициялар жана тийиштүү саясаттардын аркасында Кыргыз Республикасы неонаталдык өлүмгө учуроонун көрсөткүчтөрүн 54% чейин азайтууга жетишти. Анткен менен асфиксия менен ара төрөлүү мурдагыдай эле неонаталдык өлүмгө учуроонун башкы себептери болуп калууда, ал эми гестациялык курак үчүн кичинекей болгон (ГКК) жана ара төрөлгөн балдардын жашоосунун алгачкы айында өлүмгө учуроо тобокелдиги гестациялык куракка шайкеш келген жана убагында төрөлгөн ымыркайларга салыштырмалуу 80 эсеге жогору болууда. Бойго бүтүрбөөчү каражаттарды пайдалануудан тышкары негизги кийлигишүүлөргө камтуу көбөйдү жана, жалпысынан алганда, чектелген социалдык-демографиялык теңсиздик шартында жогорку деңгээлде калууда.

Интерпретация: Кыргыз Республикасында неонаталдык өлүмгө учуроонун көрсөткүчтөрү кыйла төмөндөдү жана бул тенденцияны андан ары да сактоого дарамет бар. Жаңы төрөлгөн ымыркайлардын жашап кетүүсү жана өлүү болуп төрөлүүнү азайтуу боюнча №3 Туруктуу Өнүгүү Максатынын милдеттерин аткаруу үчүн Кыргыз Республикасы салмагы аз жана оорукчан балдарга кам көрүү боюнча кийлигишүү топтомдорунун масштабын кеңейтип, бардык медициналык мекемелердеги кам көрүү иштеринин сапатын, перинаталдык кам көрүү боюнча регионалдык бөлүмдөрдүн болушун камсыздоого жана тез кайтарым байланышта болгон жана энелер менен ымыркайлар үчүн өз ара байланыштагы отчет берип тура турган улуттук каттоо тутумун түзүүгө тийиш.
