# Supplementary material for: Trends and determinants of newborn mortality in Kyrgyzstan: a Countdown country case study
Source: Lancet Glob Health. 2020 Dec 10;9(3):e352–60. doi: 10.1016/S2214-109X(20)30460-5 (PMC7886658; doi:10.1016/S2214-109X(20)30460-5)
Supplement: For the Russian translation [file mmc2.pdf]

THE LANCET
Global Health

Supplementary appendix 2

This translation in Russian was submitted by the authors and we reproduce it as supplied. It has not been peer reviewed. *The Lancet*'s editorial processes have only been applied to the original in English, which should serve as reference for this manuscript.

Этот перевод на русский язык был предоставлен авторами, и мы воспроизводим его в том виде, в котором он был предоставлен. Этот перевод не был рецензирован. Редакционные процессы журнала The Lancet были применены только к оригиналу на английском языке, который служит отсылкой для этой статьи.

Supplement to: Kamali M, Wright JE, Akseer N, et al. Trends and determinants of newborn mortality in Kyrgyzstan: a Countdown country case study. *Lancet Glob Health* 2020; published online Dec 10. http://dx.doi.org/10.1016/S2214-109X(20)30460-5.

Тенденции и показатели смертности новорождённых в Кыргызской Республике: Обратный отсчёт для странового тематического исследования

Махдис Камали[1], Джеймс Э. Райт[1], Надия Аксиир[1], Хана Тасик[1], Кэйтлин Конвэй[1], Саман Брар[1], Чолпон Иманалиева[2], Геррит Маритц[2], Арджуманд Ризви[3], Бахтияр Станбеков[4], Сагынбу Абдувалиева[5], Эльвира Тоялиева[6], Зульфикар А. Бхутта [1,3*]

[1] Центр глобального здоровья детей, Госпиталь для больных детей, Торонто, Канада
[2]ЮНИСЕФ, Кыргызстан
[3]Отдел по охране здоровья женщин и детей, Университет Ага Хана, Карачи, Пакистан
[4] *Центр Электронного Здравоохранения, Министерство здравоохранения, Кыргызстан*
[5] *Национальный центр охраны материнства и детства, Министерство здравоохранения, Бишкек, Кыргызстан*
[6] *Независимый эксперт по перинатальному уходу, Бишкек, Кыргызстан*

Аннотация

Предпосылки: Кыргызская Республика достигла значительного прогресса в снижении показателей детской смертности по сравнению с регионом, несмотря на сравнительно низкий ВВП на душу населения. Благодаря наличию установленной системы регистрации рождений, мы провели комплексную оценку тенденций и показателей смертности новорождённых.

Методы: Мы использовали доступные хранилища данных и уникальный единый государственный регистр новорожденных для изучения тенденций и неравенств в репродуктивном, материнском здоровье и здоровье новорождённых и смертности за период между 1990 и 2018 годами на национальном и субнациональном уровне. Охват вмешательств по охране материнского здоровья и здоровья новорождённых был оценен и дезагрегирован в разрезе аспектов равенства. Метод динамического разложения Оаксака-Блиндера был использован для определения контекстуальных факторов, связанных с наблюдаемым снижением показателей неонатальной смертности. Мы также провели комплексный обзор национальных политик и программ, а также перспективный анализ с использованием инструмента (LiST) для привлечения внимания к тем вмешательствам, которые обладают наибольшим потенциалом для спасения жизней.

Выводы: За две последние декады, благодаря ключевым инвестициям и надлежащим политикам, Кыргызская Республика добилась снижения показателей неонатальной смертности до 54%. Однако, главными причинами неонатальной смертности остаются асфиксия и недоношенность, а для недоношенных и маленьких для гестационного возраста (МГВ) новорожденных, риск умереть в первый месяц жизни возрастает в 80 раз, по сравнению с доношенными и соответствующими гестационному возрасту (СГВ) новорожденными. За исключением использования контрацептивов, охват основными вмешательствами увеличился, и в целом остаётся высоким при ограниченных социально-демографических неравенствах.

Интерпретация: В Кыргызской Республике было достигнуто значительное снижение показателей неонатальной смертности, с потенциалом для их дальнейшего сокращения. Для того, чтобы достичь и превзойти задачи Цели Устойчивого Развития №3 по выживаемости новорождённых и сокращению мертворождений, Кыргызская Республика должна расширить масштаб пакетов вмешательств по уходу за маловесными и больными детьми, обеспечив качество ухода во всех медицинских учреждениях с развитием регионализации перинатальной помощи, и создать взаимосвязанную национальную систему регистров для матерей и новорождённых с быстрой обратной связью и подотчётностью.
