# Supplementary material for: Trends and determinants of newborn mortality in Kyrgyzstan: a Countdown country case study
Source: Lancet Glob Health. 2020 Dec 10;9(3):e352–60. doi: 10.1016/S2214-109X(20)30460-5 (PMC7886658; doi:10.1016/S2214-109X(20)30460-5)
Supplement: For the French translation [file mmc3.pdf]

THE LANCET
Global Health

Supplementary appendix 3

This translation in French was submitted by the authors and we reproduce it as supplied. It has not been peer reviewed. *The Lancet*'s editorial processes have only been applied to the original in English, which should serve as reference for this manuscript.

Cette traduction en français a été proposée par les auteurs et nous l'avons reproduite telle quelle. Elle n'a pas été examinée par des pairs. Les processus éditoriaux du *Lancet* n'ont été appliqués qu'à l'original en anglais et c'est cette version qui doit servir de référence pour ce manuscrit.

Supplement to: Kamali M, Wright JE, Akseer N, et al. Trends and determinants of newborn mortality in Kyrgyzstan: a Countdown country case study. *Lancet Glob Health* 2020; published online Dec 10. http://dx.doi.org/10.1016/S2214-109X(20)30460-5.

Contexte Le Kirghizistan a fait des progrès considérables dans la réduction de la mortalité infantile par rapport à d'autres pays la région, malgré une situation économique relativement faible. Cependant, la mortalité maternelle reste élevée. Avec le disponibilité d'un système d'enregistrement des naissances établi, nous visions à évaluer globalement les tendances et déterminants de la santé reproductive, maternelle, néonatale et infantile au Kirghizistan.

Méthodologie Pour cette étude de cas nationale du Countdown to 2030, nous avons utilisé des référentiels de données accessibles au public et registre des naissances du Kirghizistan pour examiner les tendances et les inégalités en matière de santé reproductive, de santé maternelle et néonatale et de mortalité, entre 1990 et 2018, aux niveaux national et infranational. La couverture des interventions néonatales et maternelles a été évaluée et ventilée par dimensions d'équité. Nous avons fait une décomposition Oaxaca-Blinder pour déterminer les facteurs contextuels associés à la réduction observée des taux de mortalité néonatale. Nous avons également entrepris un examen complet des politiques et programmes nationaux, ainsi qu'une analyse prospective de l'outil Lives Saved Tool, pour mettre en évidence les interventions susceptibles d'éviter le plus de décès maternels, néonatals et infantiles.

Résultats Au cours des deux dernières décennies, le Kirghizistan a réduit les taux de mortalité néonatale de 46% et les taux de mortalité infantile moins de 5 ans de 66%, tandis que les taux de mortalité maternelle ont été réduits de 7% et les taux de mortinatalité de 29%. Les causes des décès néonatals étaient la prématurité et l'asphyxie ou l'hypoxie, et les prématurés petits pour l'âge gestationnel étaient plus de 80 fois plus susceptibles de mourir au cours de leur premier mois de vie par rapport aux personnes nées appropriées pour-âge gestationnel à terme. À l'exception de l'utilisation de la contraception, la couverture des interventions essentielles a augmenté et est généralement élevé, avec peu d'inégalités sociodémographiques. Avec l'intensification de quelques interventions essentielles pour les nouveau-nés et les mères, 39% des décès néonatals, 11% des mortinaissances et 19% des décès maternels pourraient être évités d'ici 2030.

Interprétation Le Kirghizistan a considérablement réduit les taux de mortalité néonatale, avec un potentiel de réduction supplémentaire. Pour atteindre et dépasser les objectif de le Sustainable Development Goal 3 pour la survie des nouveau-nés et la réduction des mortinaissances, le Kirghizistan doit étendre les ensembles d'interventions pour les soins des nouveau-nés petits et malades, garantir la qualité des soins dans tous les établissements de soins périnatals et créer un registre national lié pour les mères et les nouveau-nés avec une rétroaction rapide et une responsabilisation.
