# Supplementary material for: Trends and determinants of newborn mortality in Kyrgyzstan: a Countdown country case study
Source: Lancet Glob Health. 2020 Dec 10;9(3):e352–60. doi: 10.1016/S2214-109X(20)30460-5 (PMC7886658; doi:10.1016/S2214-109X(20)30460-5)
Supplement: For the Arabic translation [file mmc4.pdf]

THE LANCET
Global Health

Supplementary appendix 4

This translation in Arabic was submitted by the authors and we reproduce it as supplied. It has not been peer reviewed. *The Lancet*'s editorial processes have only been applied to the original in English, which should serve as reference for this manuscript.

تم تقديم هذه الترجمة باللغة العربية من قبل المؤلفين ونعيد إنتاجها كما هو مُقدم. إنها لم تخضع لاستعراض الأقران. تم تطبيق عمليات
تحرير لانسيت فقط على النص الأصلي باللغة الإنجليزية، والذي يجب أن يكون بمثابة مرجع لهذه المخطوطة.

Supplement to: Kamali M, Wright JE, Akseer N, et al. Trends and determinants of newborn mortality in Kyrgyzstan: a Countdown country case study. *Lancet Glob Health* 2020; published online Dec 10. http://dx.doi.org/10.1016/S2214-109X(20)30460-5.

الخلفية: أحرزت قيرغيزستان تقدمًا كبيرًا في الحد من وفيات الأطفال مقارنة ببلدان أخرى في المنطقة ، على الرغم من الوضع الاقتصادي المتدني نسبيًا. ومع ذلك ، لا تزال وفيات الأمومة مرتفعة. نظرًا لتوافر نظام راسخ لتسجيل المواليد ،هدفنا إجراء تقييم شامل لاتجاهات ومحددات الصحة الإنجابية وصحة الأم والوليد والطفل في قيرغيزستان.

الطرق: في هذه الدراسة للحالة القطرية من منظمة ” كونتدون تو 2030” ، استخدمنا البيانات المتاحة للعامةوسجلات المواليد الوطنية في قيرغيزستان لفحص الاتجهات وعدم المساواة في الصحة الإنجابية وصحة الأم والوليد والوفيات بين عامي 1990 و 2018 ، على المستوى الوطني و المناطقي تم تقييم تغطية تدخلات صحة المواليد والأمهات وتصنيفها حسب أبعاد مختلفة . أجرينا تحليل “أواكساكا - بلايندر” لتحديد العوامل المرتبطة بالانخفاض الملحوظ في معدلات وفيات حديثي الولادة. كما أجرينا مراجعة شاملة للسياسات والبرامج الوطنية ، بالإضافة إلى تحليل محتمل “ليفس سايفد تول”، لتسليط الضوء على التدخلات التي لديها القدرة على تجنب معظم وفيات الأمهات والمواليد والأطفال.

الموجودات: على مدى العقدين الماضيين ، خفضت قيرغيزستان معدلات وفيات الأطفال حديثي الولادة بنسبة ٪46 ومعدلات وفيات الأطفال الذين تقل أعمارهم عن 5 سنوات بنسبة 66٪ ، في حين انخفضت معدلات وفيات الأمهات بنسبة 7٪ ومعدلات الإملاص بنسبة 29٪ [أ: إضافة النتائج الأخرى ، ومعدل المواليد الموتى ينخفض٪ ، طيب؟]. كانت الأسباب الرئيسية لوفيات الأطفال حديثي الولادة هي الخداج والاختناق أو نقص الأكسجين ، وكان الأطفال الخدج وصغيري الحجم بالنسبة لسن الحمل أكثر عرضة للوفاة بأكثر من 80 مرة في الشهر الأول من حياتهم مقارنة بمن ولدوا عند أوانهم و بحجم مناسب لسن الحمل .باستثناء استخدام موانع الحمل ، زادت تغطية المداخلات الأساسية وهذه التغطية مرتفعة بشكل عام ، مع قلة عدمالمساواة الاجتماعية والديموغرافية . إن مع توسيع نطاق بعض المداخلات الأساسية للأطفال حديثي الولادة والأمهات ، يمكن منع 39٪ من وفيات حديثي الولادة ، و 11٪ من حالات الإملاص ، و 19٪ من وفيات الأمهات بحلول عام .2030

التأويل: خفضت قيرغيزستان معدلات وفيات الأطفال حديثي الولادة إلى حد كبير ، مع احتمال حدوث مزيد من الانخفاض. لتحقيق أهداف “التنمية المستدامة 3” وتجاوزها من أجل بقاء المواليد الجدد على قيد الحياة والحد من حالات الإملاص ، تحتاج قيرغيزستان إلى توسيع نطاق باقة المداخلات لرعاية الأطفال الصغار والمرضى ، وضمان جودة الرعاية في جميع مرافق الرعاية الصحية مع رعاية ما قبل الولادة في الأقاليم ، وإنشاء سجل وطني مرتبط بالأمهات وحديثي الولادة مع كل المراجعات والمسؤليات المترتبة .
