# Supplementary material for: Trends and determinants of newborn mortality in Kyrgyzstan: a Countdown country case study
Source: Lancet Glob Health. 2020 Dec 10;9(3):e352–60. doi: 10.1016/S2214-109X(20)30460-5 (PMC7886658; doi:10.1016/S2214-109X(20)30460-5)
Supplement: For the Spanish translation [file mmc5.pdf]

THE LANCET
Global Health

Supplementary appendix 5

This translation in Spanish was submitted by the authors and we reproduce it as supplied. It has not been peer reviewed. *The Lancet*'s editorial processes have only been applied to the original in English, which should serve as reference for this manuscript.

Los autores nos proporcionaron esta traducción al español y la reproducimos tal como nos fue entregada. No la hemos revisado. Los procesos editoriales de *The Lancet* se han aplicado únicamente al original en inglés, que debe servir de referencia para este manuscrito.

Supplement to: Kamali M, Wright JE, Akseer N, et al. Trends and determinants of newborn mortality in Kyrgyzstan: a Countdown country case study. *Lancet Glob Health* 2020; published online Dec 10. http://dx.doi.org/10.1016/S2214-109X(20)30460-5.

Introducción Kirguistán ha logrado avances considerables en la reducción de la mortalidad infantil en comparación con otros países de la región, a pesar de una situación económica comparativamente baja. Sin embargo, la mortalidad materna sigue siendo alta. Con el disponibilidad de un sistema de registro de nacimientos establecido, nuestro objetivo era evaluar de manera integral las tendencias y los determinantes de la salud reproductiva, materna, neonatal e infantil en Kirguistán.

Métodos Para este estudio de caso de país de Countdown to 2030, utilizamos repositorios de datos disponibles al público y registro de nacimientos de Kirguistán para examinar las tendencias y desigualdades de la salud reproductiva, la salud materna, neonatal y la mortalidad, entre 1990 y 2018, a nivel nacional y subnacional. Se evaluó la cobertura de las intervenciones neonatales y maternas y se desglosó por dimensiones de equidad. Hicimos la descomposición Oaxaca-Blinder para determinar los factores contextuales asociados con la reducción observada en las tasas de mortalidad neonatal. También llevamos a cabo una revisión exhaustiva de las políticas y programas nacionales, así como un análisis prospectivo de la herramienta Vidas salvadas, para destacar las intervenciones que tienen el potencial de evitar la mayoría de las muertes maternas, neonatales e infantiles.

Recomendaciones Durante las últimas dos décadas, Kirguistán redujo las tasas de mortalidad de recién nacidos en un 46% y las tasas de mortalidad infantile menores de 5 años en un 66%, mientras que las tasas de mortalidad materna se redujeron en un 7% y las tasas de mortinatos en un 29%. Las causas de las muertes neonatales fueron la prematuridad y la asfixia o la hipoxia, y los prematuros pequeños para la edad gestacional tenían más de 80 veces más probabilidades de morir en su primer mes de vida en comparación con los nacidos a término apropiados para la edad gestacional. A excepción del uso de anticonceptivos, la cobertura de las intervenciones esenciales ha aumentado y, en general, es alta, con pocas inequidades sociodemográficas. Con la ampliación de algunas intervenciones esenciales para el recién nacido y la madre, el 39% de las muertes neonatales, el 11% de los mortinatos y el 19% de las muertes maternas podrían prevenirse para 2030.

Interpretación Kirguistán ha reducido considerablemente las tasas de mortalidad de recién nacidos, con potencial para una mayor reducción. Para lograr y superar las metas del objetivo de Sustainable Development Goal 3 para la supervivencia de los recién nacidos y la reducción de la mortinatalidad, Kirguistán necesita ampliar los paquetes de intervenciones para la atención de los recién nacidos pequeños y enfermos, garantizar la calidad de la atención en todos los establecimientos de salud con regionalizado atención perinatal y crear un registro nacional vinculado para madres y recién nacidos con rápida retroalimentación y responsabilidad.
