# Supplementary material for: Trends and determinants of newborn mortality in Kyrgyzstan: a Countdown country case study
Source: Lancet Glob Health. 2020 Dec 10;9(3):e352–60. doi: 10.1016/S2214-109X(20)30460-5 (PMC7886658; doi:10.1016/S2214-109X(20)30460-5)
Supplement: Supplementary appendix [file mmc6.pdf]

THE LANCET
Global Health

Supplementary appendix 6

This appendix formed part of the original submission and has been peer reviewed. We post it as supplied by the authors.

Supplement to: Kamali M, Wright JE, Akseer N, et al. Trends and determinants of newborn mortality in Kyrgyzstan: a Countdown country case study. *Lancet Glob Health* 2020; published online Dec 10. http://dx.doi.org/10.1016/S2214-109X(20)30460-5.

Appendix- Newborn Mortality in the Kyrgyz Republic

Table 1: Basic contextual, demographic and health service indicators for WHO's Central Asia countries

	**Population Density**	**HDI (2017)**	**Rural Population (% of total pop'n)**	**GDP per capita, PPP**	**Poverty headcount ratio at national poverty lines (% of pop'n)**	**Out-of-pocket expenditure (% of total health expenditure)**	**Government health expenditure (% of GDP)**	**Contraceptive use, modern methods**	**ANC4+**
**Kazakhstan**	6.7	0.80	46.8	24,055	2.7	38.9	1.94	49.5	95.3
**Kyrgyzstan**	32.3	0.68	64.0	3,393	25.4	48.2	2.35	38.5	94.6
**Tajikistan**	64.3	0.65	73.0	2,896	31.3	63.1	2.10	25.7	52.5
**Turkmenistan**	12.3	0.70	49.2	16,389	NA	71.1	1.54	46.0	96.4
**Uzbekistan**	76.1	0.71	63.4	6,253	14.1	42.7	2.77	59.3	78.5
**World**	58.0	NA	45.3	15,469	NA	18.1	5.89	56.1	N/A

Table 2: Breakdown of study indicators and data sources

Domain	Indicator	Source
Newborn mortality	Neonatal mortality rate, NMR<br>Number of newborns dying in the first 28 days of life per 1,000 livebirths	United Nations Inter-agency for Child Mortality Estimation (UN-IGME) (5)<br>Institute for Health Metrics and Evaluation (IHME) (10)<br>DHS (15,16)<br>MICS (12-14)<br>National birth registry
Stillbirth	Stillbirth rate, SBR<br>Number of stillbirths per 1,000 births	IHME (10)<br>National birth registry
Newborn cause of death	Leading causes of death for newborns in first 28 days of life (ICD-10)	Child Health Epidemiology Reference Group (CHERG) (17)<br>National birth registry<br>UN-IGME (5)
Live births	The complete extraction from its mother of a product of conception, after such separation, breathing or other evidence of life (beating heart, pulsation of umbilical cold, or movement of voluntary muscles) is shown	United Nations Population Division (18)
Cause of stillbirth	Leading causes of stillbirth (ICD-10)	National birth registry
Maternal Mortality	Maternal death is the death of a woman while pregnant or within 42 days of termination of pregnancy (per 100,000 live births)	United Nations Maternal Mortality Estimate Interagency Group (UN-MMEIG) (11)
Maternal conditions	Underlying maternal conditions amongst women whose infants die in the first 28 days of life (ICD-10)	National birth registry
Maternal and newborn health interventions	Coverage of continuum of care interventions aimed at mothers and newborns	DHS (15,16)<br>MICS (12-14)

*Mortality estimates for other Central Asian countries are taken from UN-IGME (for NMR and U5MR) and UN-MMEIG (for MMR)

Table 3: Number of births according to official estimates and births registered, 2013-2017

Year	Number of births according to UN-Pop estimates (17)	Number of births registered in the birth registry	Difference (%)
2013	153637	128859	-
2014	154483	156001	0.98
2015	153977	157721	2.43
2016	154800*	150997	2.45
2017	154800*	154742	0.04

*Average annual number of births calculated by dividing the projected total for 5-year period (2016-2020)

Figure 1: Number of registered births by oblast

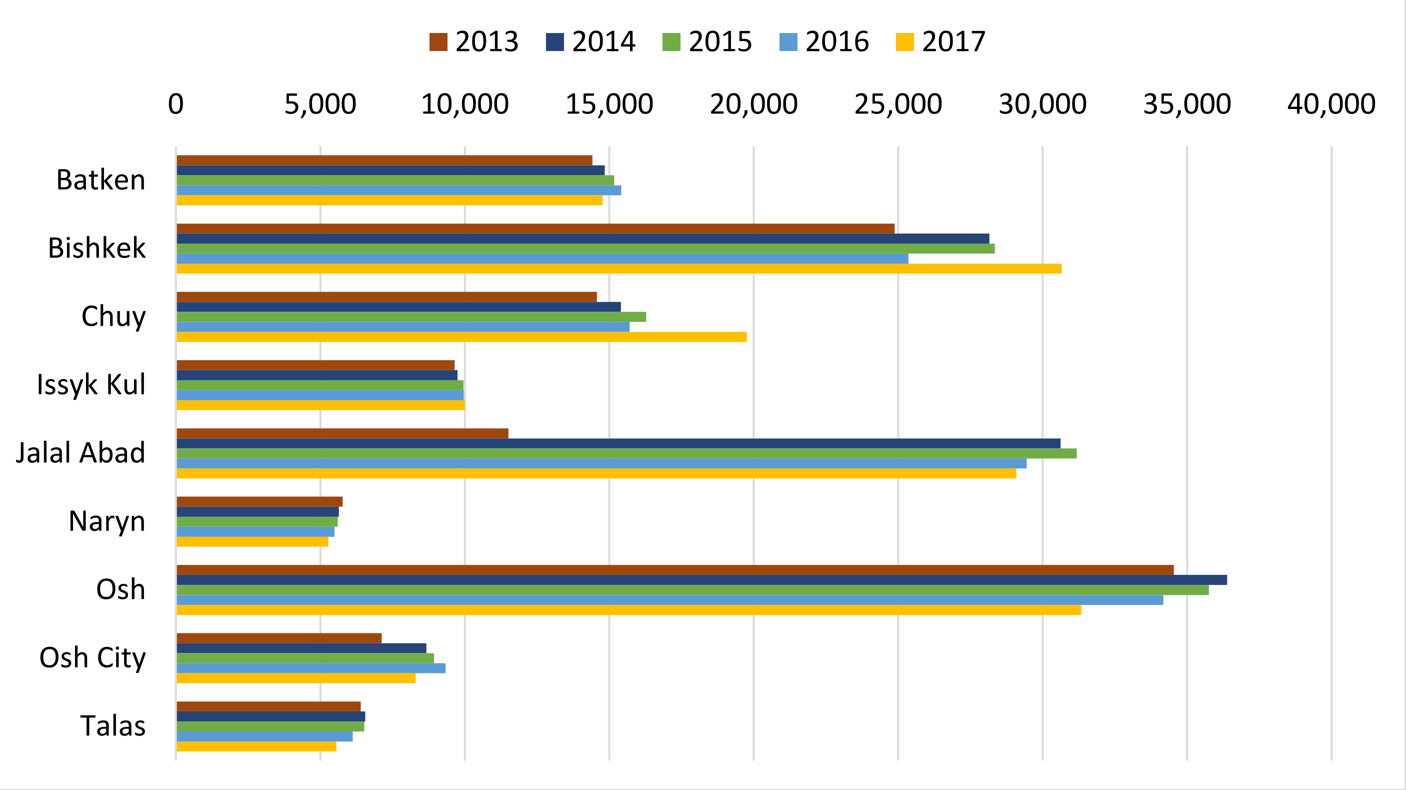

Figure 2: Number of registered deaths by oblast

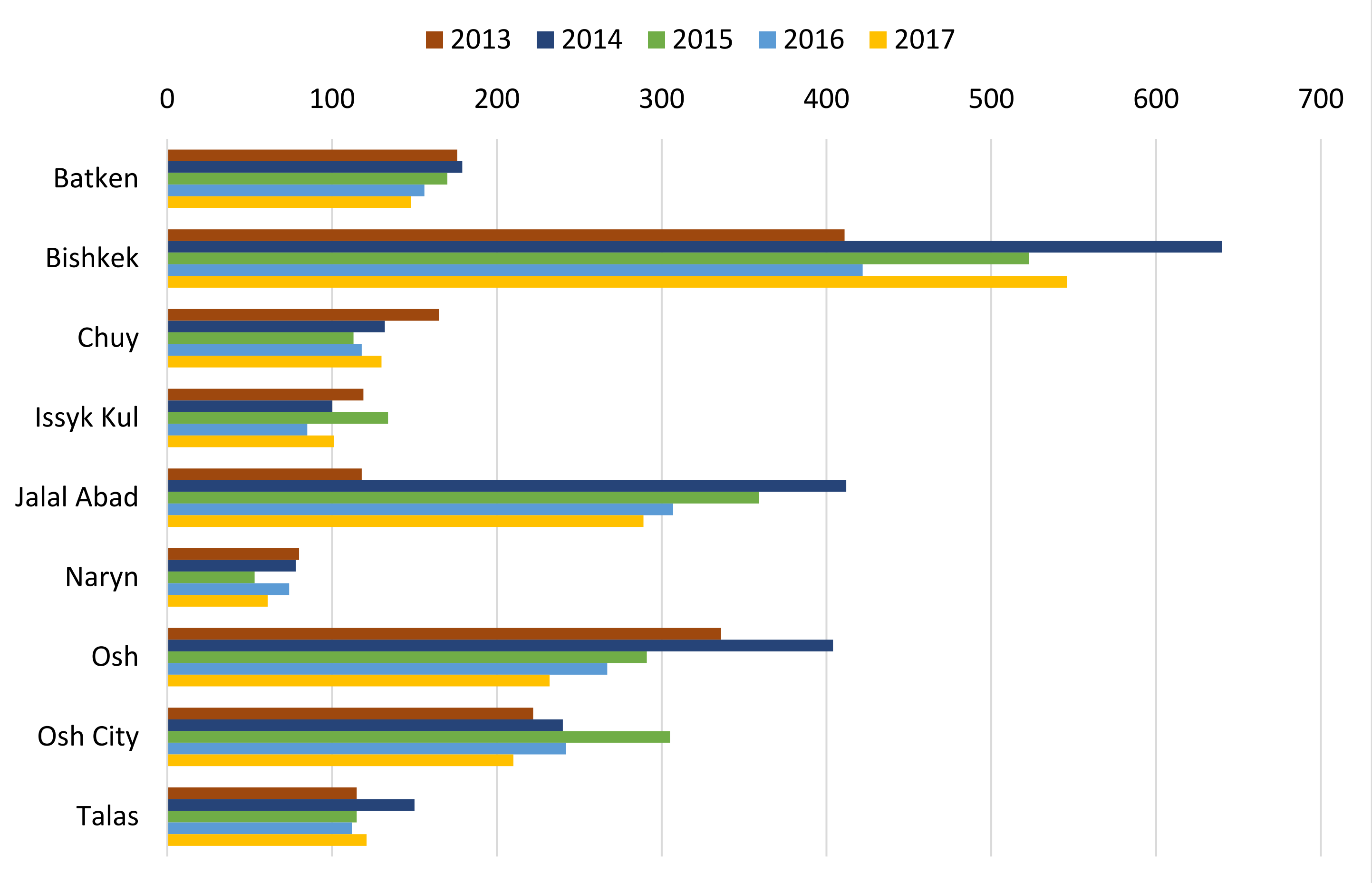

Table 4: Indicators and definition of essential interventions across the continuum of care

Stage	Indicator	Definition
CCI	Composite coverage index	Provides an overall estimate of coverage. A weighted average of: demand for family planning satisfied, antenatal care 1+ visits, skilled birth attendance, Baccille Calmette Guérin vaccine, 3 doses of diphtheria, pertussis, tetanus vaccine, measles vaccination, Oral rehydration with continued feeding for diarrhea treatment, and care seeking for suspected pneumonia
Family planning	Contraceptive prevalence (modern and traditional methods)	Prevalence of current contraceptive use among married women 15-49 years old, any method (%)

	Demand for family planning satisfied	Percentage of total demand for family planning among married or in-union women aged 15 to 49 that is satisfied (contraceptive prevalence divided by total demand for family planning)
Maternal and newborn care	Antenatal care 1+ visit with a skilled provider	Percentage of women attended at least once during pregnancy by skilled health personnel
	Antenatal care 4+ visits with any provider	Percentage of women attended four or more times during pregnancy by any provider
	Skilled birth attendance	Percentage of live births attended by skilled health personnel
	Health facility-based deliveries	Percentage of women given birth in a health facility
	Women delivering via caesarean section	Percentage of women who delivered via
	Early initiation of breastfeeding	Percentage of newborns put to the breast within one hour of birth
	Exclusive breastfeeding 0-5 months of age	Exclusive breastfeeding refers to the percentage of children less than six months old who are fed breast milk alone (no other liquids) in the past 24 hours
Postnatal care	Postnatal care for all babies	Percentage of newborns who received postnatal care within two days of birth
	Postnatal care for mothers	Percentage of mothers who received postnatal care within two days of childbirth

Figure 3: Conceptual framework of newborn survival (1)

A conceptual framework regarding newborn survival was created to separate variables into one of three levels corresponding to distal, intermediate, and proximal determinants of newborn mortality.

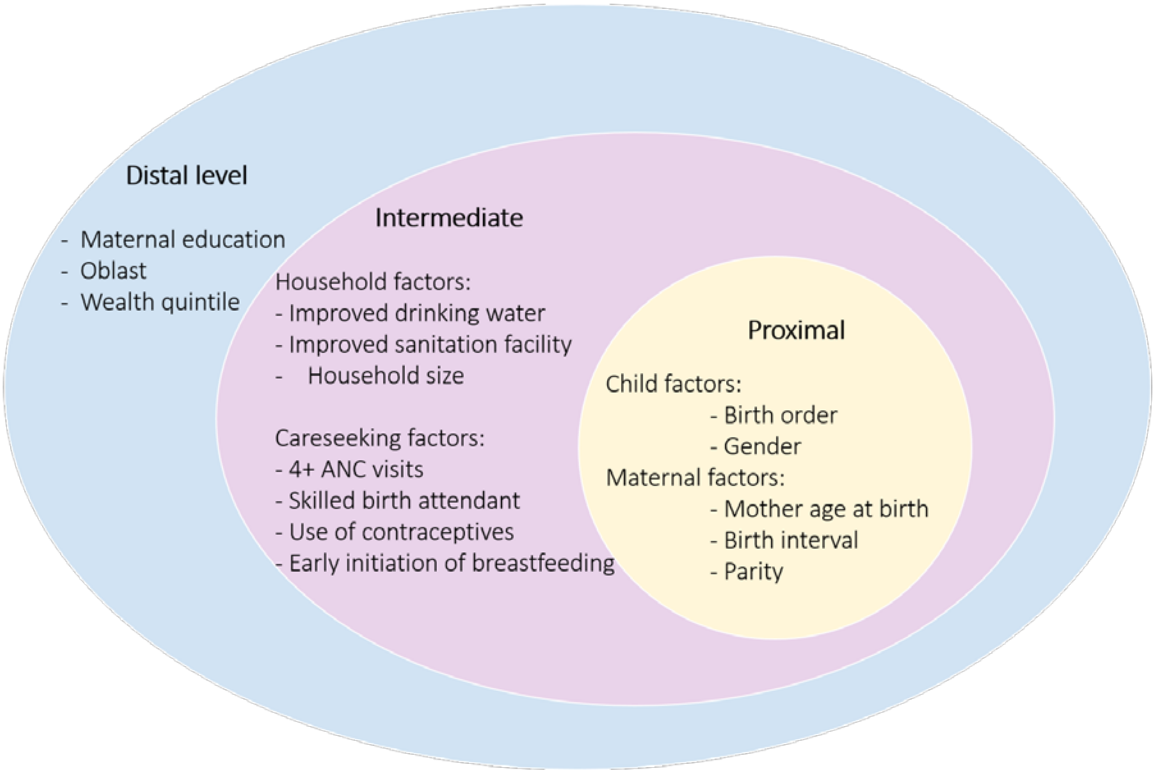

Figure 4: MMR in Central Asia region and Kyrgyzstan

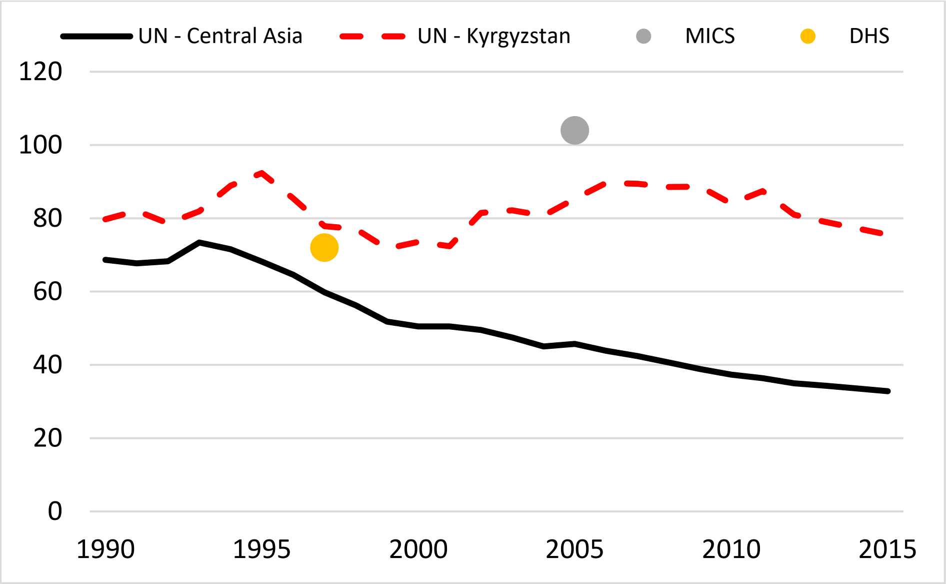

Figure 5: Stillbirth trends, 1990-2017

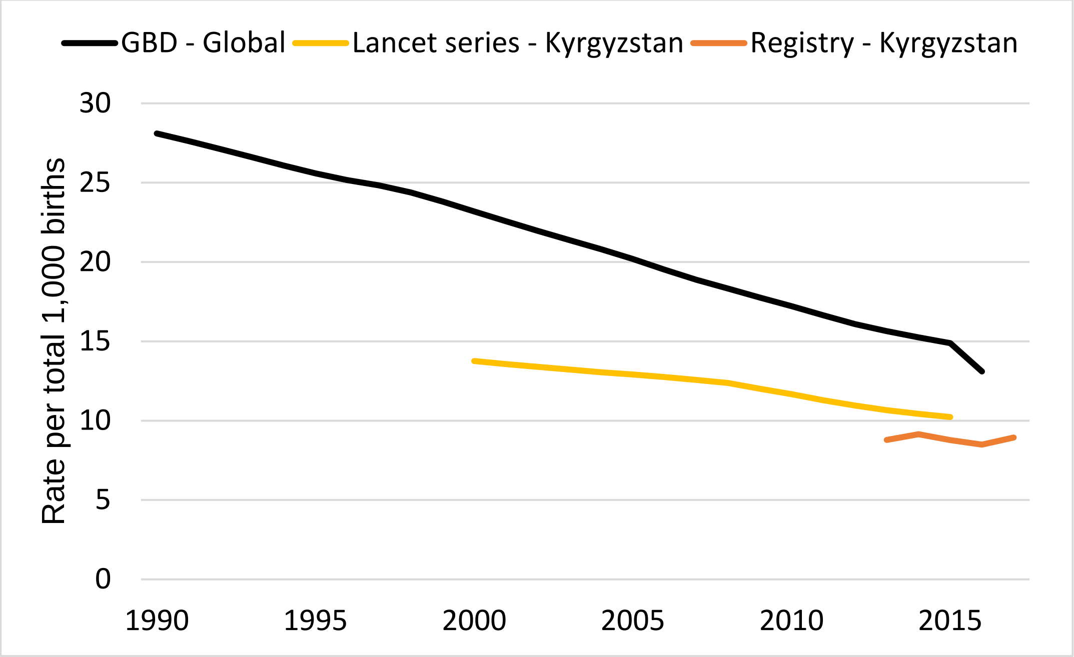

Figure 6: Under-five mortality rate in Central Asia region and Kyrgyzstan

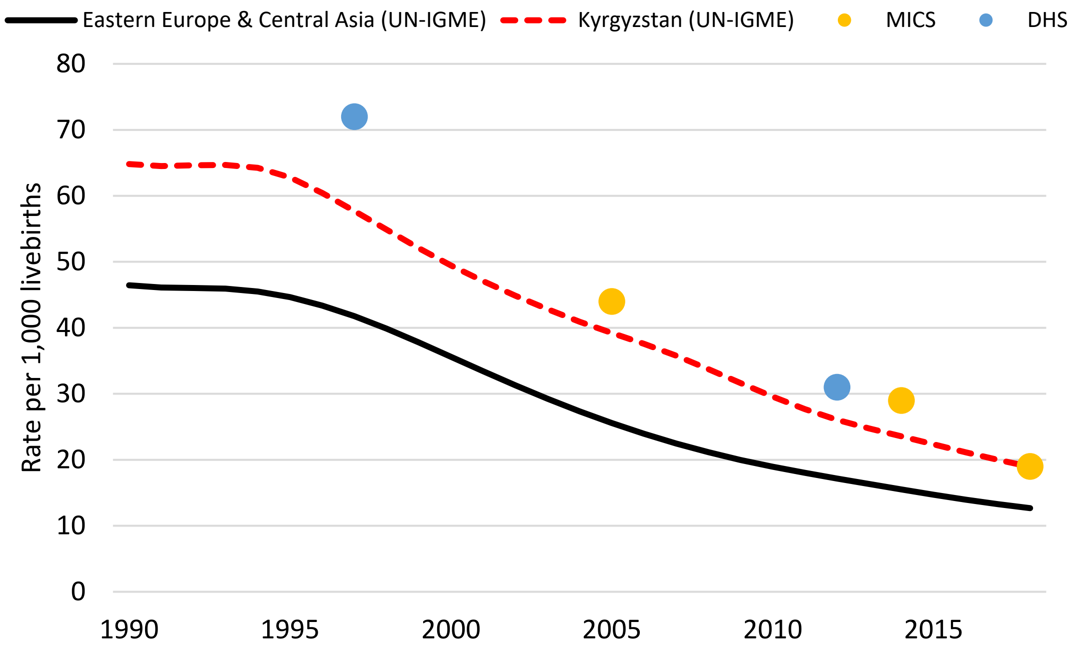

Figure 7: Neonatal mortality rate in Central Asia region and Kyrgyzstan

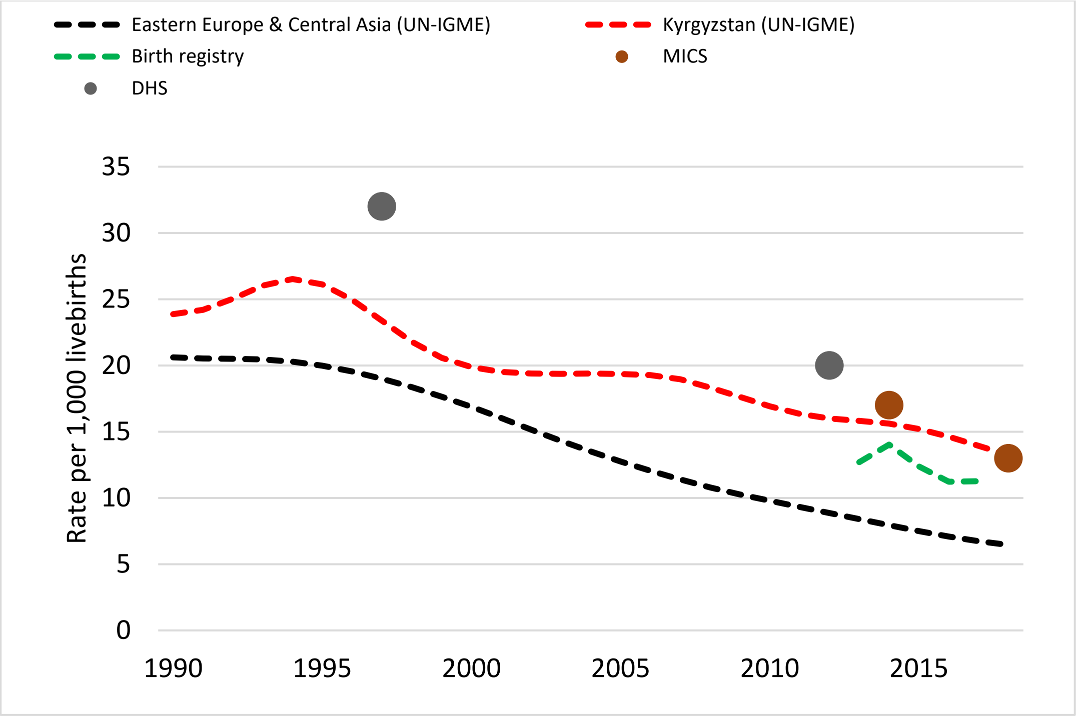

Figure 8: Oblast-level NMR trends, 2013-2017

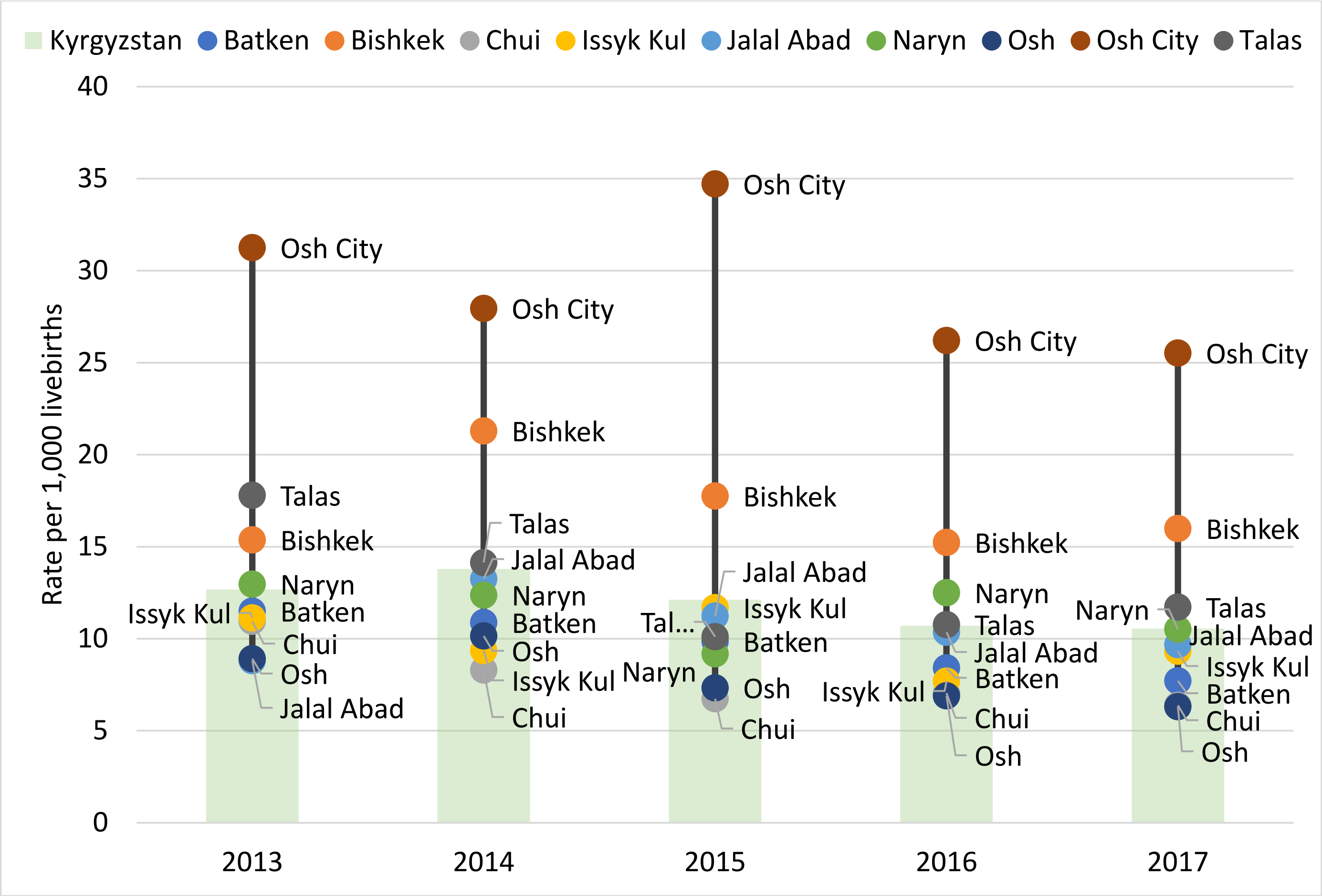

Kyrgyzstan
Batken
Bishkek
Chui
Issyk Kul
Jalal Abad
Naryn
Osh
Osh City
Talas
Rate per 1,000 livebirths
0
5
10
15
20
25
30
35
40
2013
2014
2015
2016
2017
Osh City
Talas
Bishkek
Naryn
Issyk Kul
Batken
Chui
Osh
Jalal Abad
Osh City
Bishkek
Talas
Jalal Abad
Naryn
Batken
Osh
Issyk Kul
Chui
Osh City
Bishkek
Jalal Abad
Issyk Kul
Tal...
Batken
Naryn
Osh
Chui
Osh City
Bishkek
Naryn
Talas
Jalal Abad
Issyk Kul
Batken
Chui
Osh
Osh City
Bishkek
Naryn
Talas
Jalal Abad
Issyk Kul
Batken
Chui
Osh

Figure 9: A) Rayon-level in NMR in 2017 B) Oblast-level annual rate of decline in NMR 1997-2018

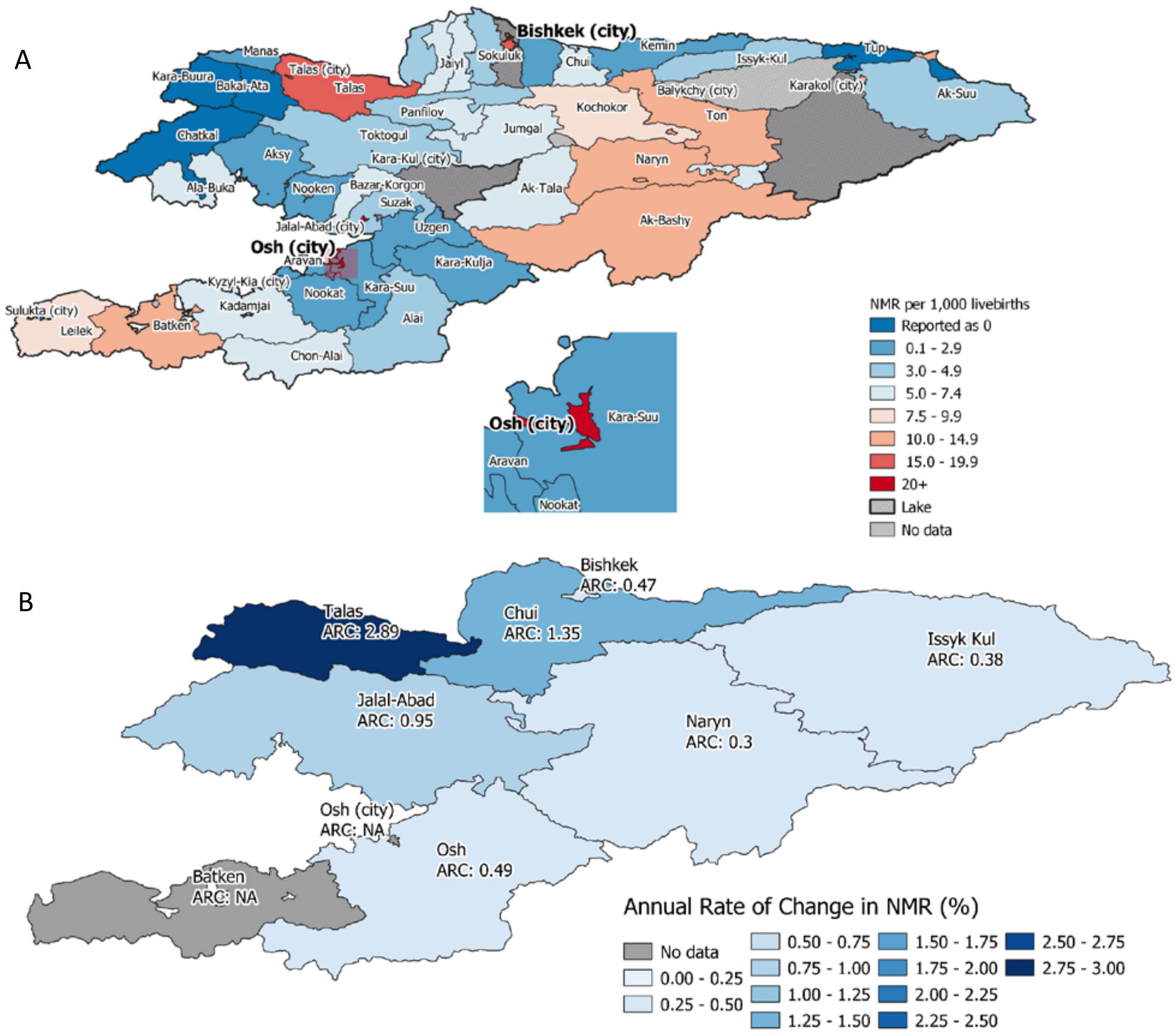

Figure 10: NMR in DHS 1997 (top) and MICS 2018 (bottom)

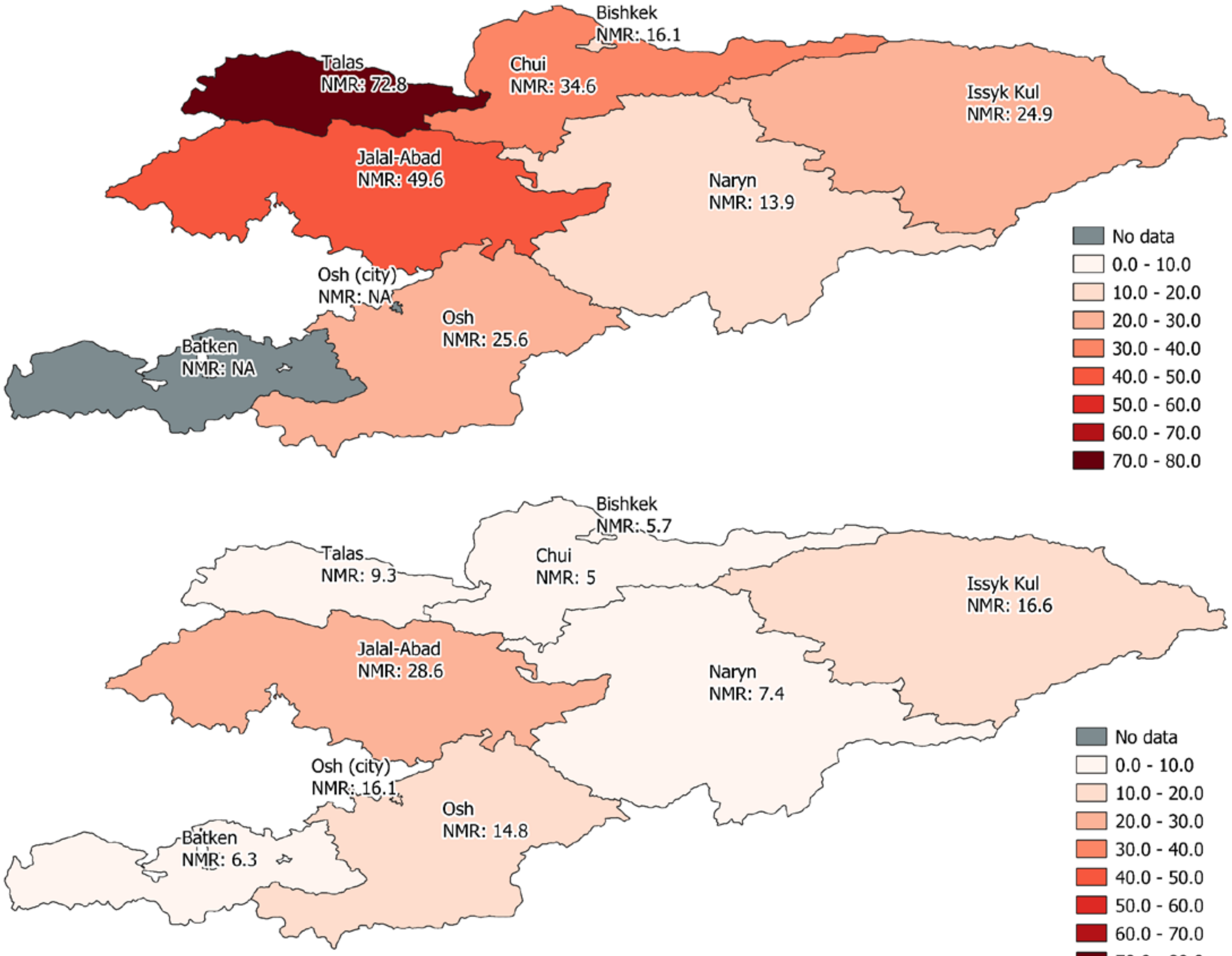

Figure 11: Equiplots of NMR by equity dimensions for DHS 1997, DHS 2012, MICS 2018

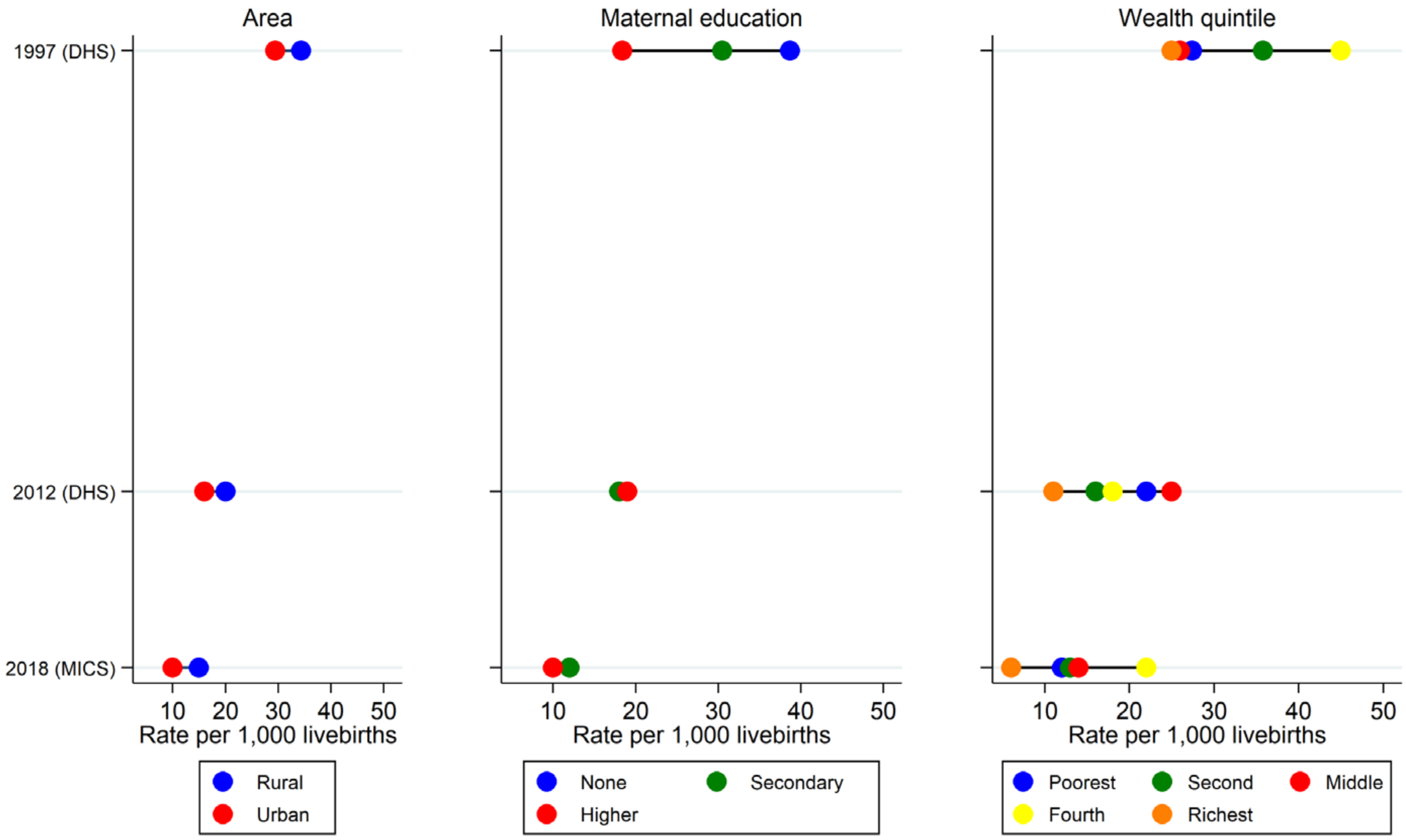

Figure 12: Comparison of neonatal and maternal intervention coverage by wealth quintile, DHS 1997 versus MICS 2014 and MICS 2018

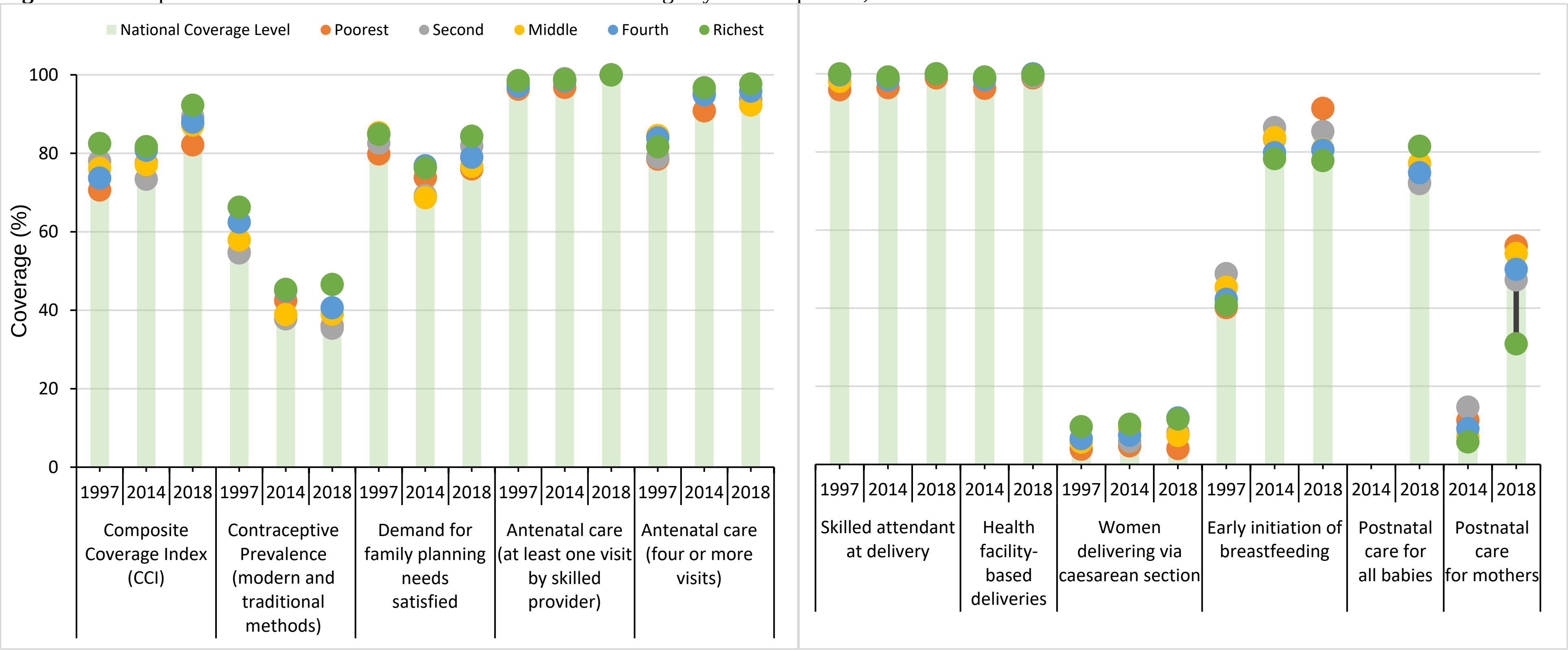

Figure 13: Comparison of neonatal and maternal intervention coverage by area of residence, DHS 1997 versus MICS 2014 and MICS 2018

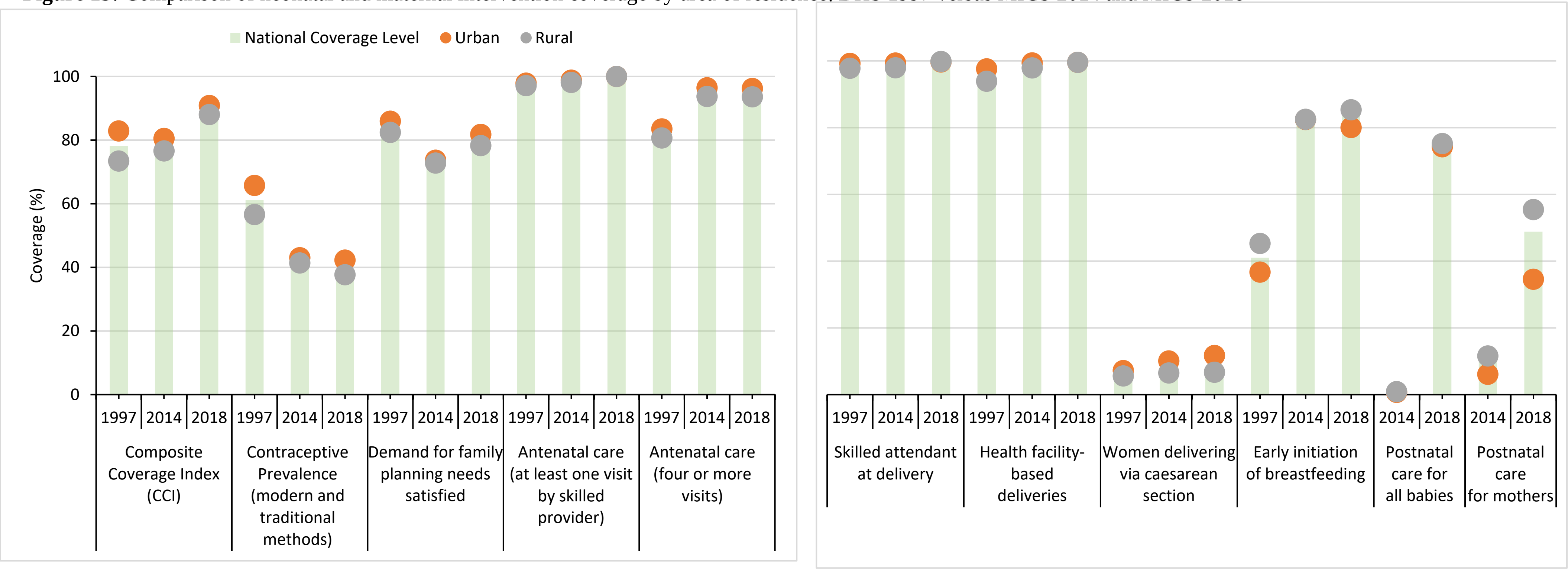

Figure 14: Quality of care interventions, 2005-2018

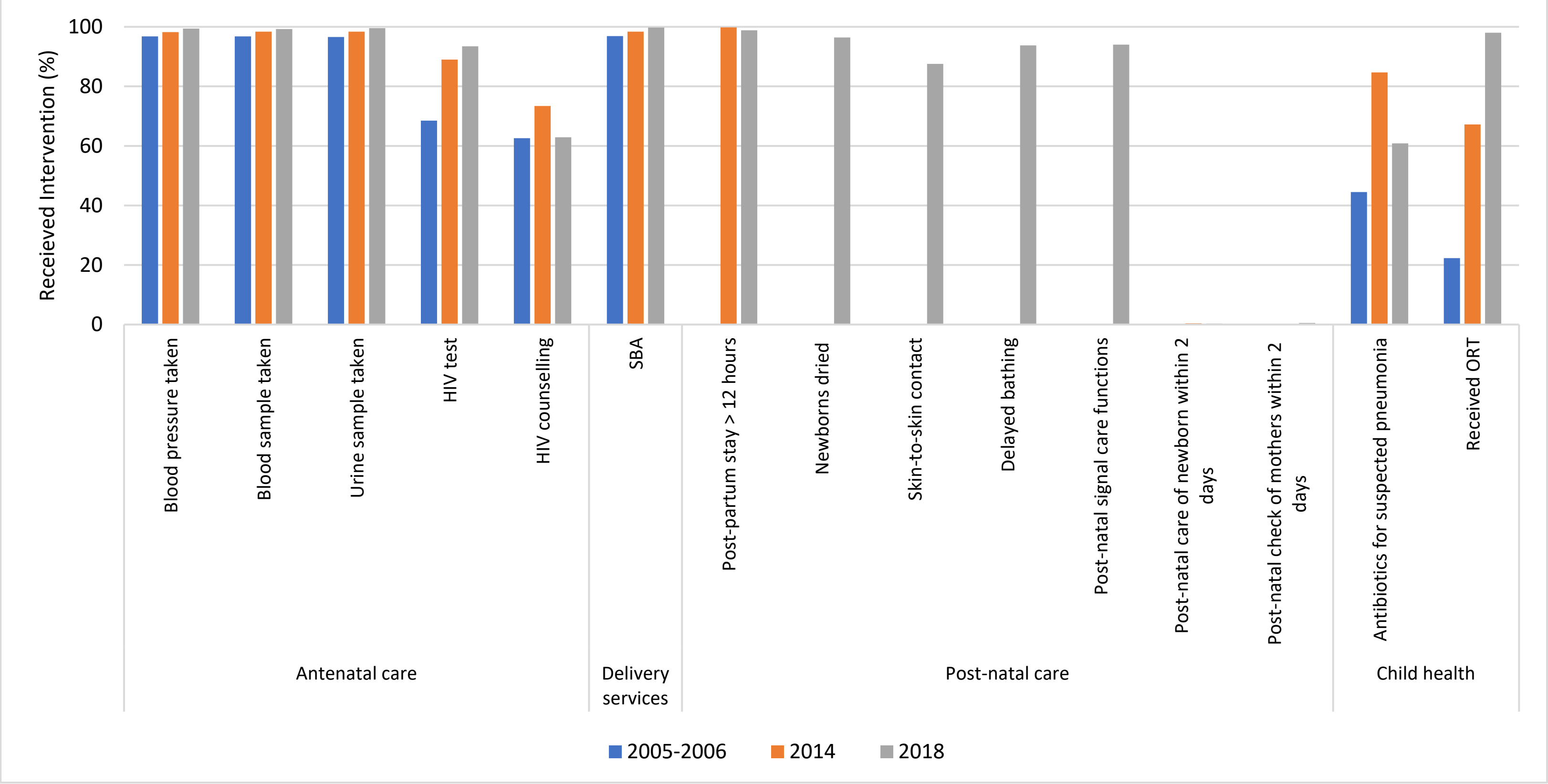

Figure 15: Percentage of mother's receiving post-natal checks post-discharge from health facility & location of the checks

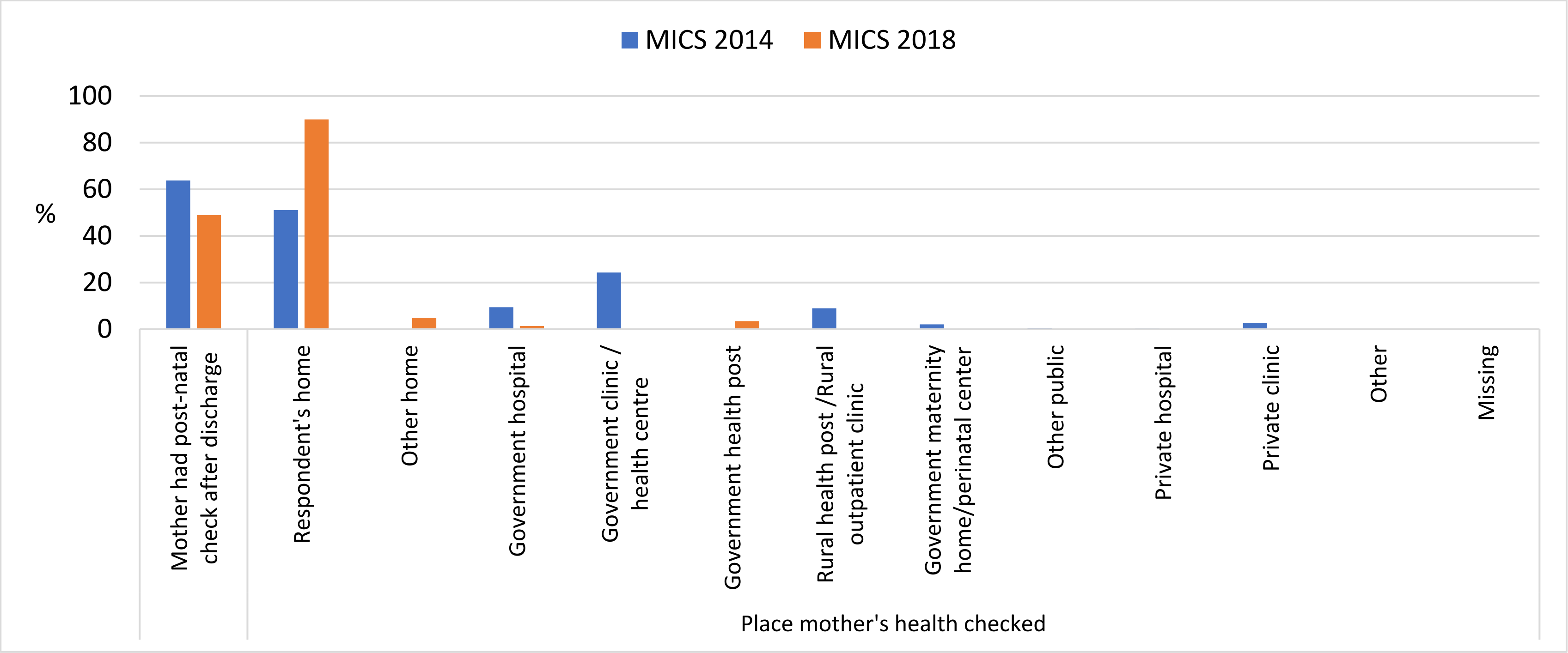

Figure 16: Leading causes of neonatal death in the Kyrgyz Republic according to National birth registry (2013-2017), CHERG (2017) and IHME-GBD (2017) estimates

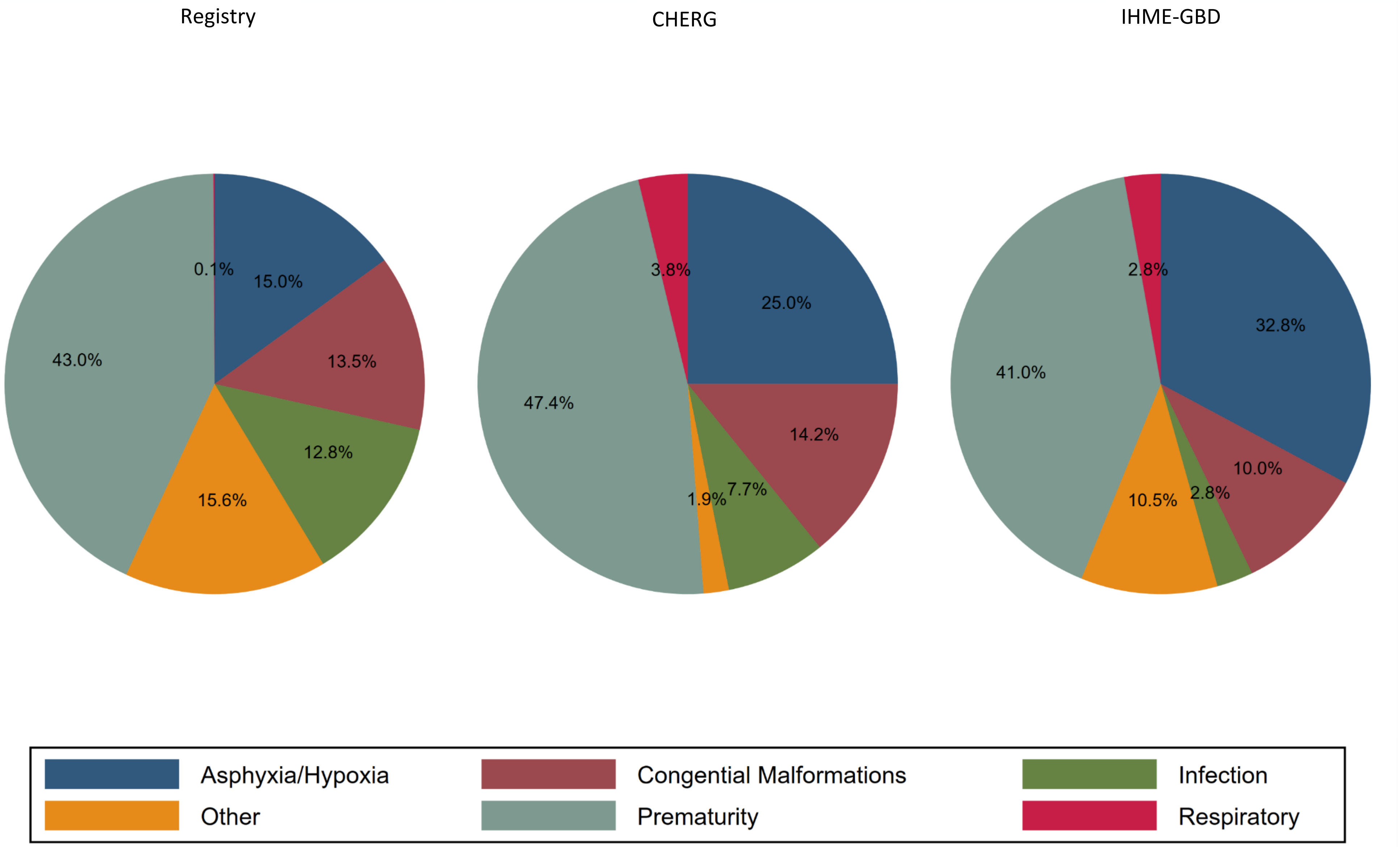

Figure 17: Leading causes of neonatal death among normal birthweight (≥2500g) and low birthweight (<2500g) infants in Kyrgyzstan, 2013-2017 (birth registry)

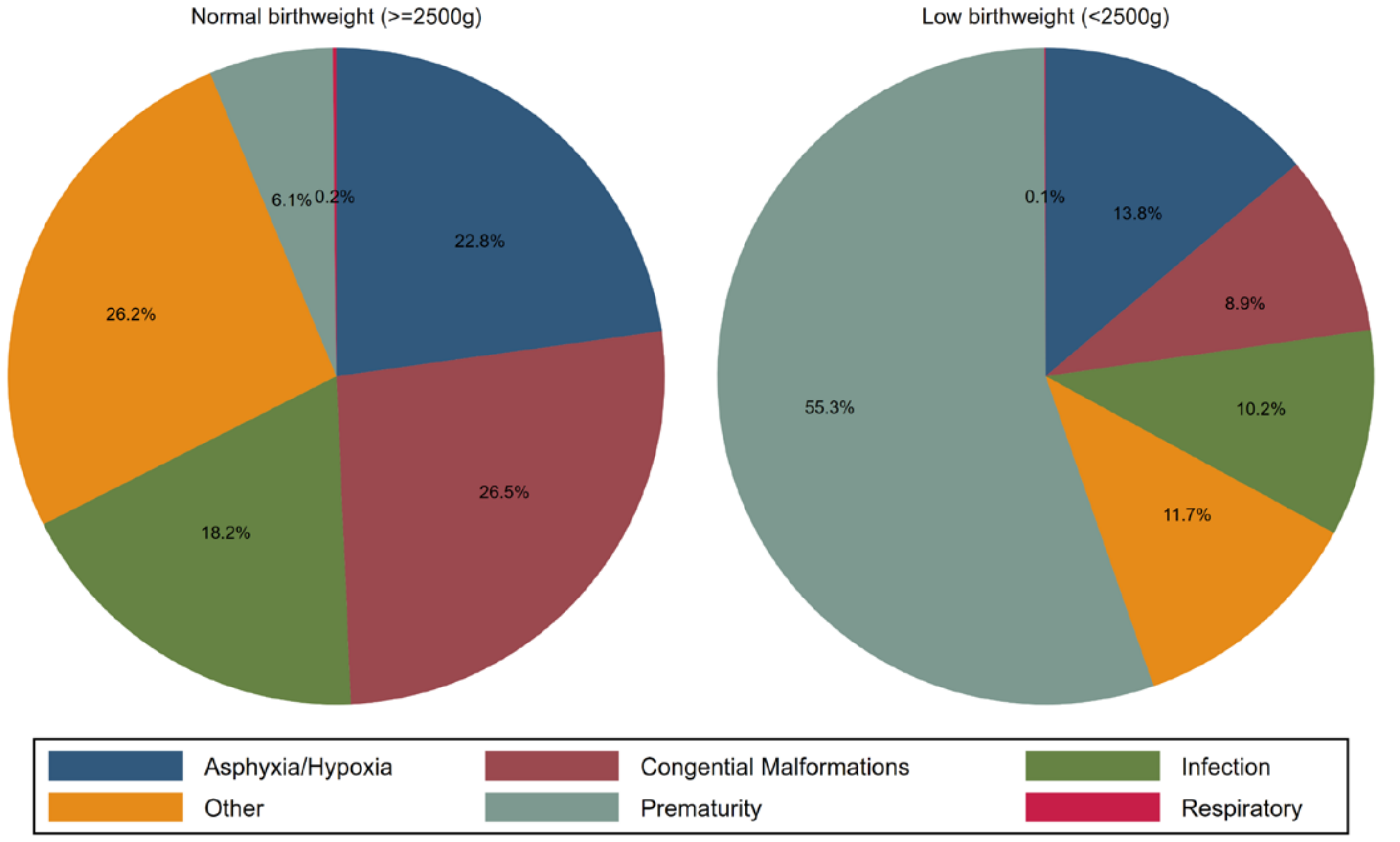

Figure 18: Leading causes of death among full-term (≥37 weeks' gestation) and pre-term (<37 weeks' gestation) infants, 2013 – 2017 (birth registry)

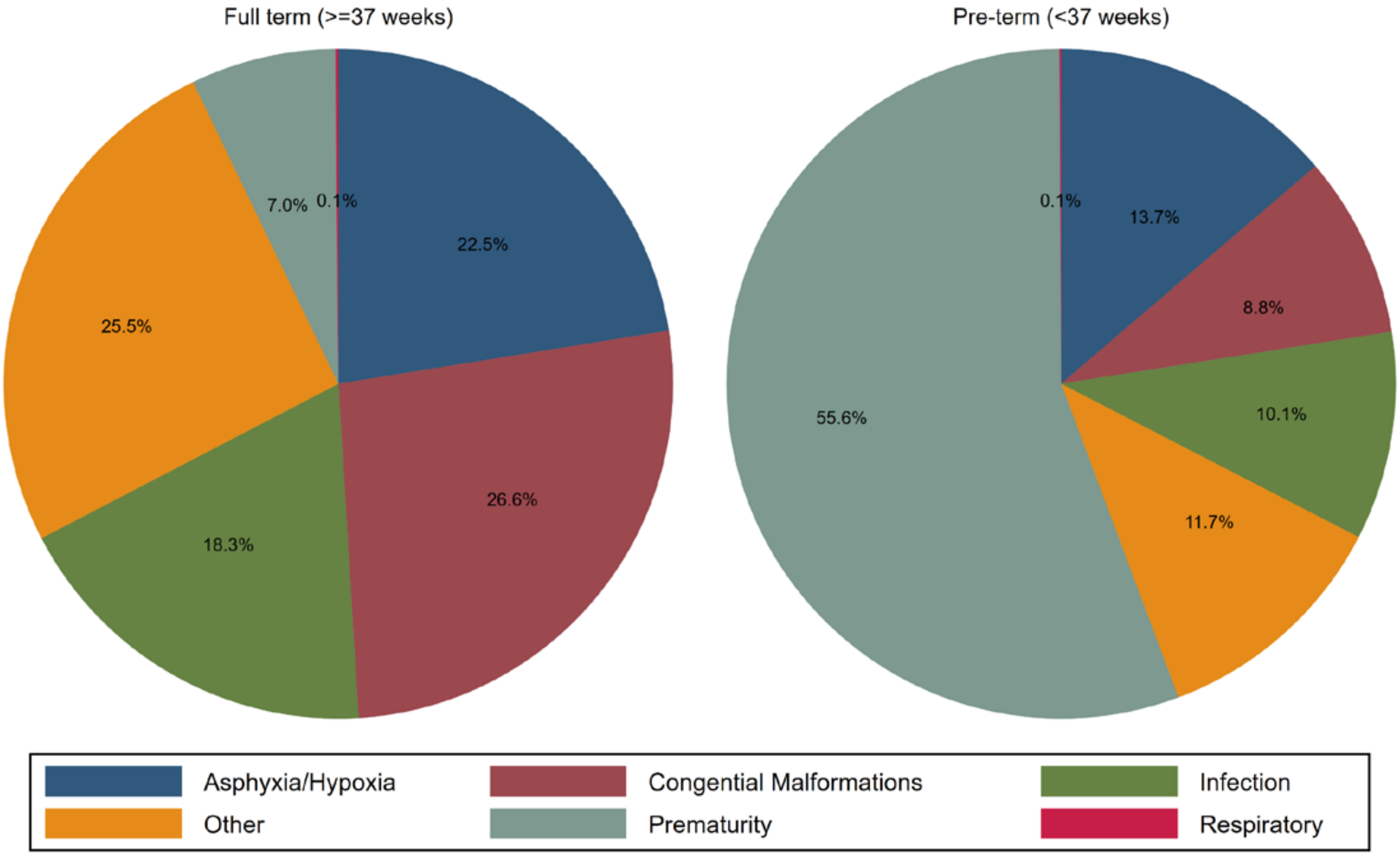

Figure 19: Leading causes of death among antenatal and intrapartum stillbirths, 2013 – 2017 (birth registry)

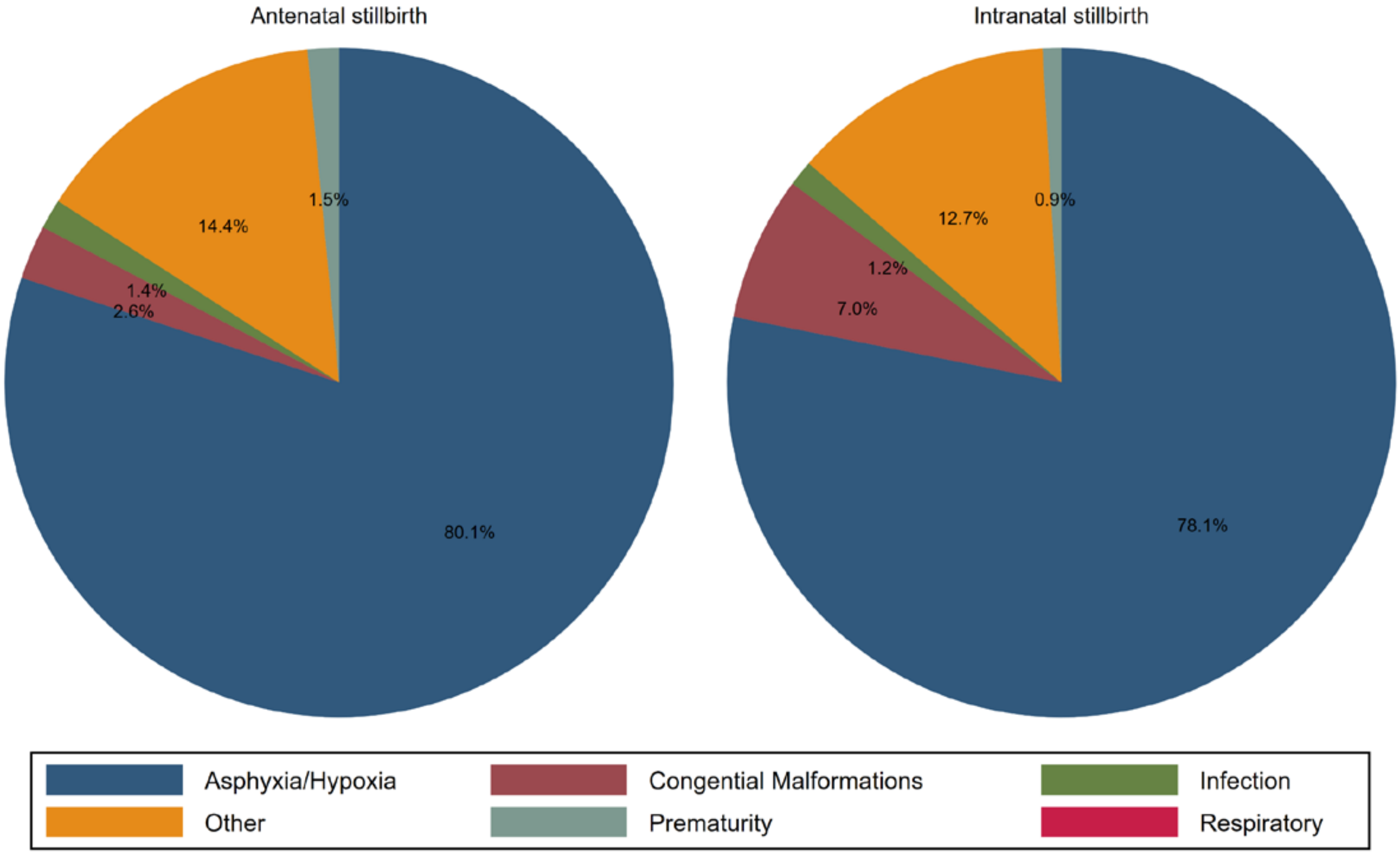

Figure 20: Timing of newborn death by day, 2013-2017 (birth registry)

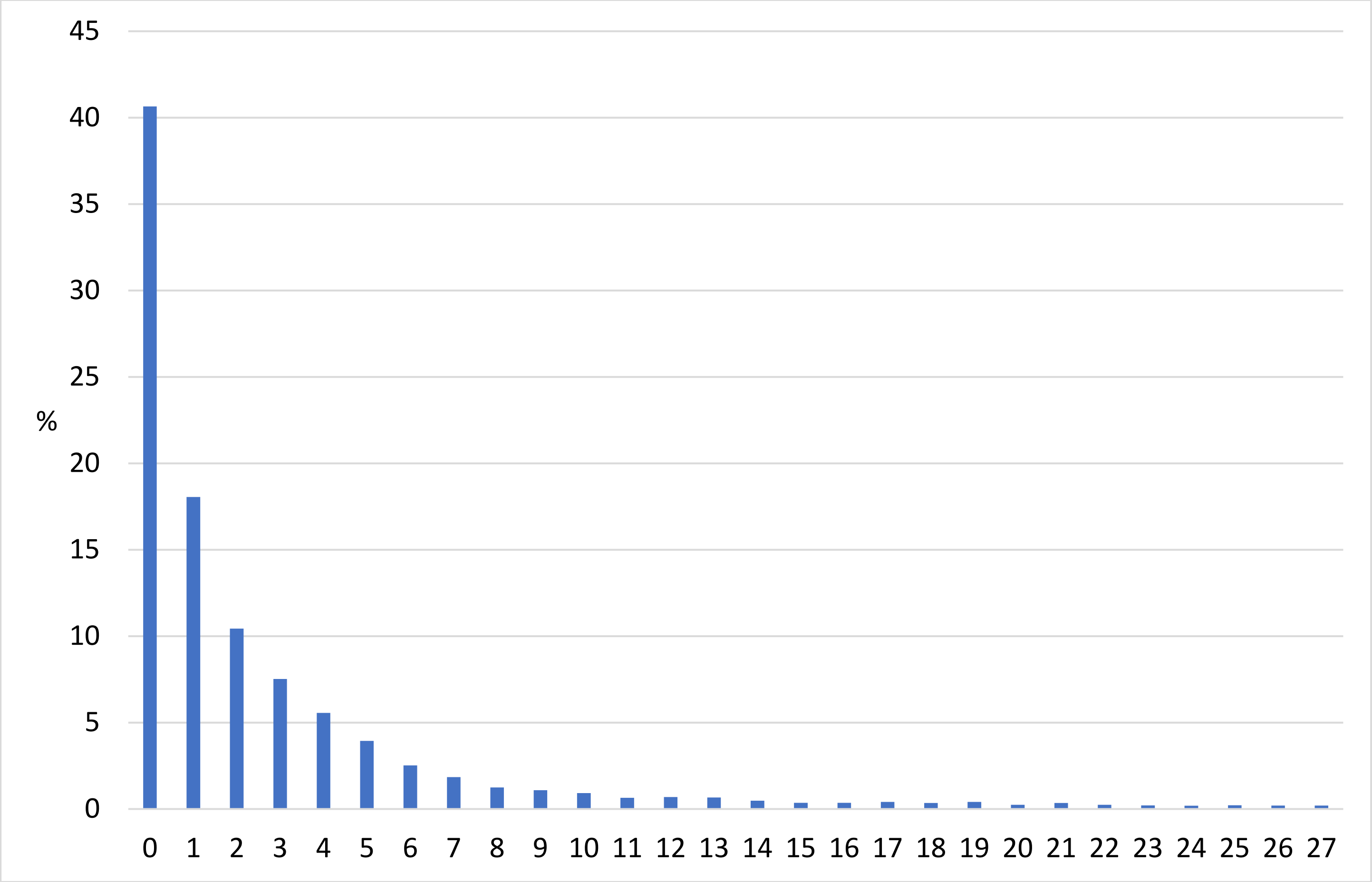

Figure 21: Breakdown of stillbirths by timing, 2013-2017 (birth registry)

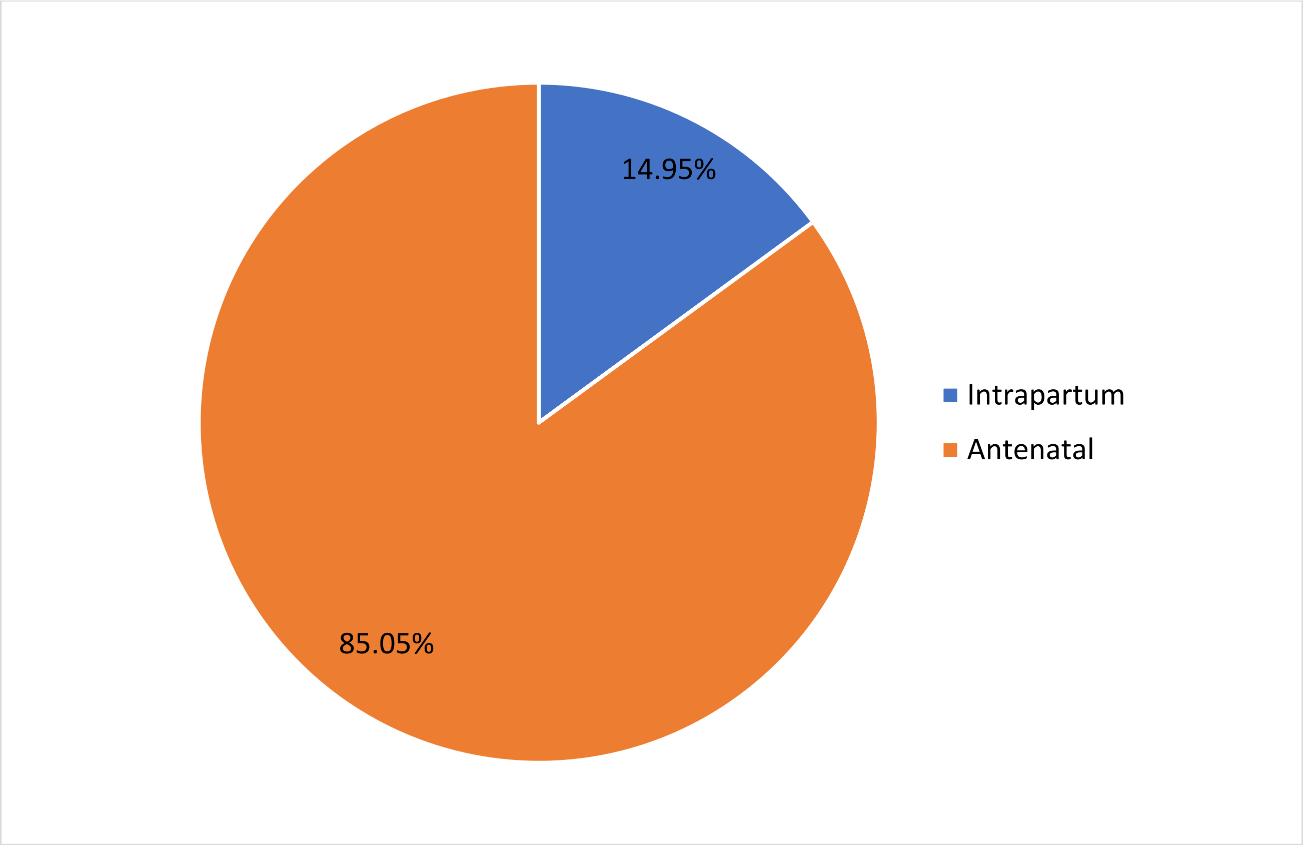

Figure 22: Neonatal mortality risk ratio by birth outcome, all deaths 2013-2017

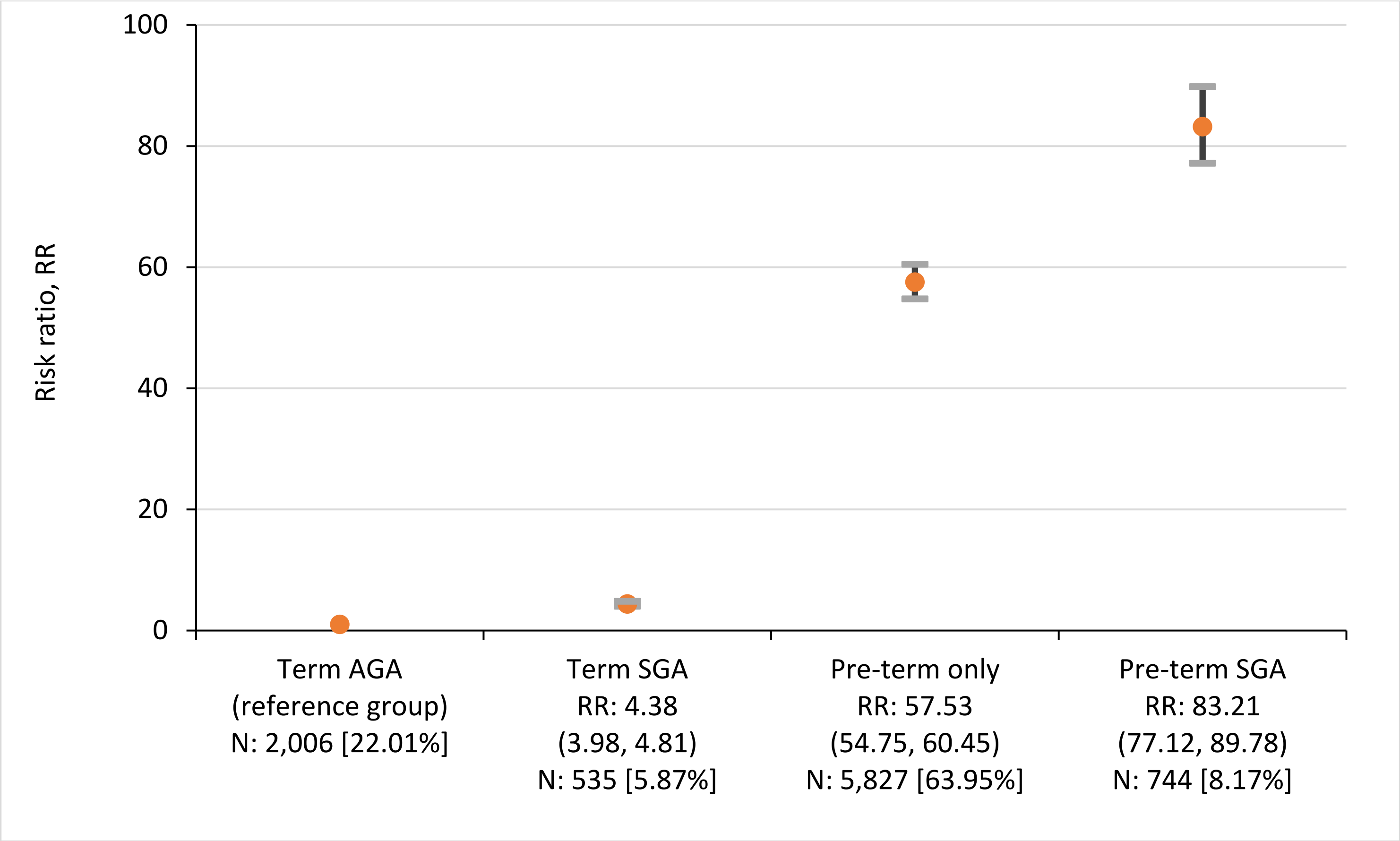

Risk ratio, RR
100
80
60
40
20
0
Term AGA (reference group) N: 2,006 [22.01%]
Term SGA RR: 4.38 (3.98, 4.81) N: 535 [5.87%]
Pre-term only RR: 57.53 (54.75, 60.45) N: 5,827 [63.95%]
Pre-term SGA RR: 83.21 (77.12, 89.78) N: 744 [8.17%]

Table 5: Factors associated with decline of NMR in Kyrgyzstan, MICS 2018 & DHS 1997

Indicators	Proportions		Change in proportions	Model (2018)		NMR by change in proportions
	1997	2018	2018-1997	Coef.	p-value	2018-1997
PROXIMAL						
Birth order (ref: First birth)						
Second	0.2708	0.2892	0.0184	0.0579	0.892	
Third	0.1945	0.1799	-0.0146	-0.2711	0.620	
Fourth +	0.2419	0.1529	-0.0890	-1.3688	0.051	0.1218
Child sex (ref: Male)						
Female	0.4972	0.4911	-0.0061	-0.6477	0.020	0.0040
Mother's age at birth (ref: 20-29)						
<20	0.0942	0.0681	-0.0261	0.5467	0.193	
30-39	0.2628	0.1845	-0.0783	0.5114	0.275	
40+	0.0164	0.0125	-0.0039	2.4728	0.020	-0.0096
Birth interval						
Mean	23.2147	28.7687	5.5541	-0.0133	0.147	-0.0737
Parity						
Mean	4.4185	3.6137	-0.8048	0.3720	<0.001	-0.2994
					**SUM**	**-0.2569**
					**exp(sum)=rel.risk**	**0.7734**
INTERMEDIATE						
Household size (ref: Less than 7 people)						
7+ people	0.4690	0.4552	-0.0138	-0.4608	0.098	0.0064
Early initiation of breastfeeding (ref: Yes)						
No	0.5873	0.1492	-0.4381	0.9671	0.001	-0.4237
Improved drinking water (ref: Yes)						
No	0.2429	0.0904	-0.1525			
Improved sanitation facilities (ref: Yes)						
No	0.0042	0.0021	-0.0021			
At least four ANC visits (ref: Yes)						
No	0.1854	0.0631	-0.1223			
Skilled birth attendant at delivery (ref: Yes)						
No	0.0149	0.0022	-0.0127			
Currently using birth control (ref: Yes)						
No	0.4538	0.5663	0.1125			
					**SUM**	**-0.4173**
					**exp(sum)=rel.risk**	**0.6588**
DISTAL						
Maternal education (ref: Higher)						
None/Primary/Secondary	0.8777	0.7232	-0.1545			
Wealth index quintile (ref: Poorest)						
Second	0.2081	0.2283	0.0202			
Middle	0.1992	0.1932	-0.006			
Fourth	0.1955	0.1935	-0.002			
Richest	0.136	0.132	-0.004			
Oblast (ref: Osh)						
Bishkek	0.0693	0.1313	0.0620	0.4204	0.407	
Chui	0.1656	0.1378	-0.0278	-0.0812	0.880	
Issyk-Kul	0.0872	0.0697	-0.0175	0.6710	0.104	-0.0117
Jalal-Abad	0.1968	0.2050	0.0082	0.9919	0.017	0.0081
Naryn	0.0667	0.0443	-0.0224	0.3883	0.451	
Talas	0.0289	0.0568	0.0279	0.3353	0.543	
					**SUM**	**-0.0036**
					**exp(sum)=rel.risk**	**0.9964**
					**Overall SUM**	**-0.6778**
					**exp(sum)=rel.risk**	**0.5077**

NB: Only statistically significant variables contribute to decomposition

Figure 23: Relative contribution of each factor to observed decrease in neonatal mortality (DHS 1997, MICS 2018)

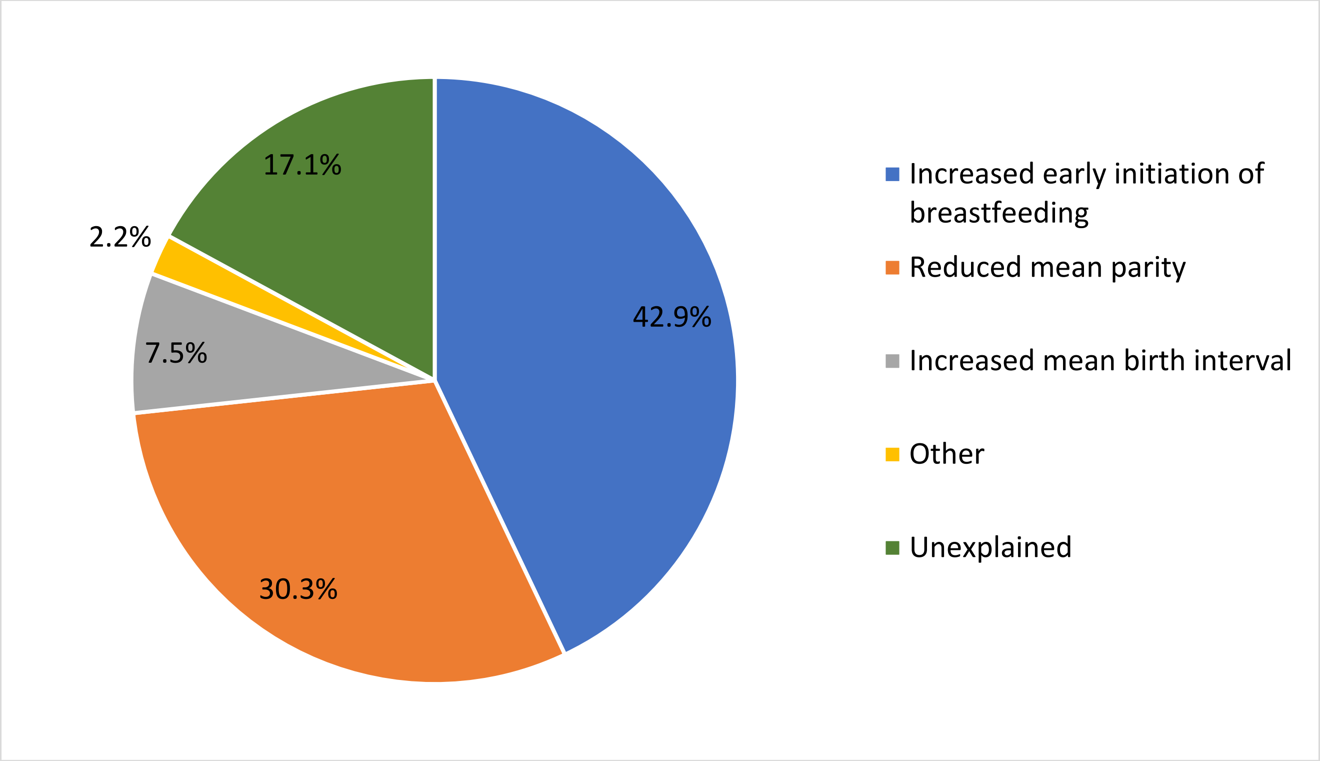

Figure 24: Laws, policies, programs and enablers related to newborn mortality from 1990-2018

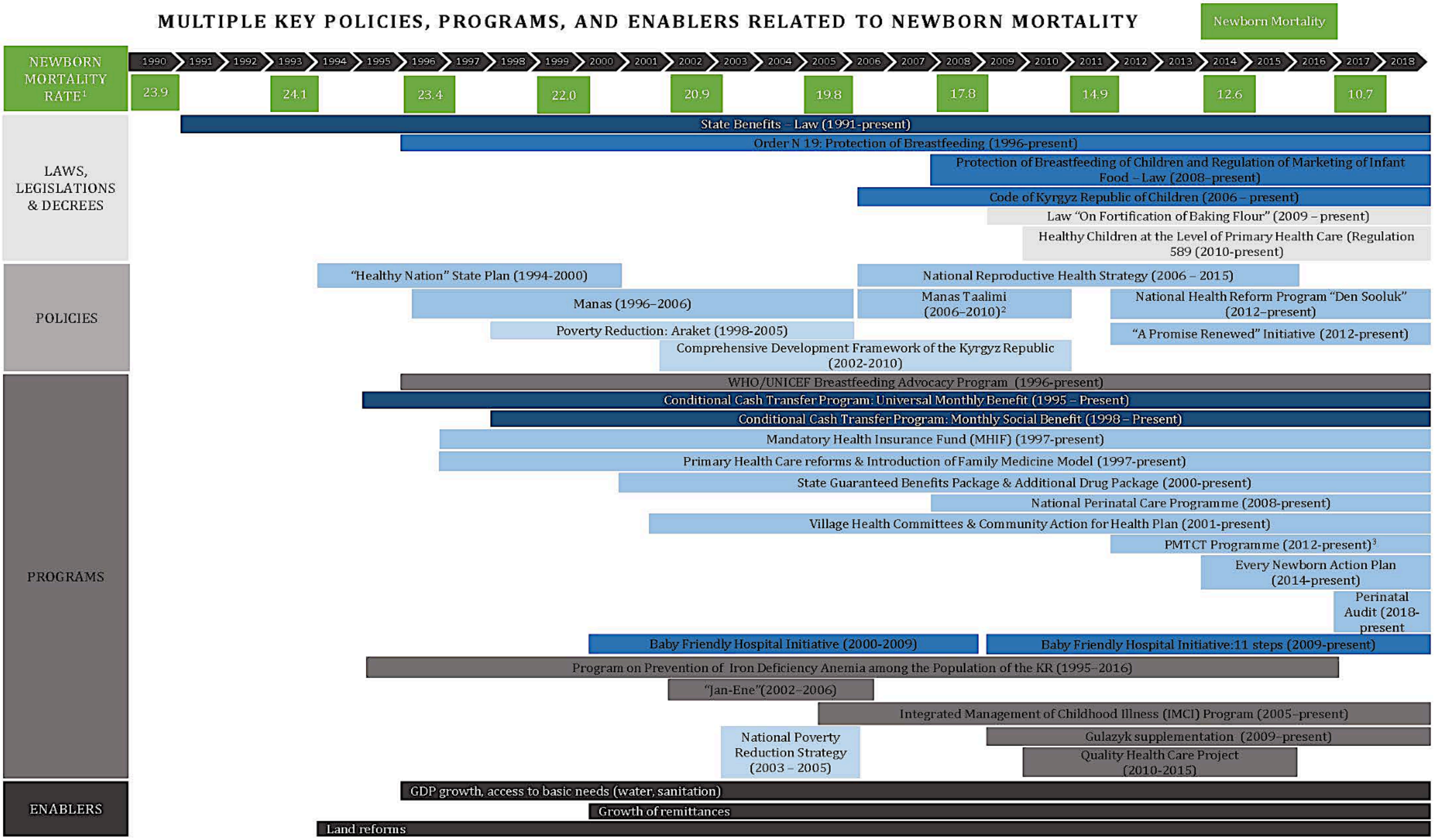

¹Estimates obtained from Unicef Global Databases ²Policy ended in 2010, but was implemented until Den Sooluk ³This programme is a part of Den Sooluk.

Policy and Program Description

i. Laws, Legislations, Decrees

State Benefits – Law (1991 – present)
This law aimed to reduce poverty among the extremely poor, families taking care of children with disabilities, orphans, and the elderly. This law led to the establishment of two cash transfer programs: the Universal Monthly Benefit (UMB) and the Monthly Social Benefit (MSB). The UMB was implemented in 1991 and focused on low income families with children <16 years of age, or <21 years of age if they were in school. The MSB was introduced in 1998 and was intended for people with disabilities, elderly, orphans, and children born to mothers with HIV (2–4).

Order N 19: Protection of Breastfeeding (1996 – present)
Established from a UNICEF/WHO advocacy campaign, this law was implemented to improve breastfeeding practices by increasing the proportion of mothers who breastfed their children. It also sought to improve perinatal care. Community health workers were tasked with dissemination of information about breast feeding to the community with a focus on pregnant women and women of reproductive age. The importance of breastfeeding was stressed, and information was provided about early initiation of breastfeeding, and the timing and duration of breastfeeding (5).

Protection of Breastfeeding of Children and the Regulation of Marketing of Infant Food (2008 – present)
This law promotes good breastfeeding practices by regulating the marketing of breastmilk substitutes. Prior to this law, medical professionals were promoting the sale of breastmilk substitutes, which are now under strict labelling requirements with prohibitions from marketing using graphics, photographs, and drawings. Breastmilk substitutes are also required to state the benefits of breastfeeding. The technical regulation of this law was introduced in 2012, and remains in practice (6). This law led to the inclusion of an 11th step to the Baby Friendly Hospital Initiative, preventing the use of breastmilk substitutes, and artificial feeding techniques.

Code of Kyrgyz Republic of children (2006 – present)
The code of Kyrgyz Republic of Children establishes the rights, freedoms and legal interests of children, including ensuring children's right to healthcare, and outlining the duties of state bodies responsible for health care. The code tasks public health institutions with protecting children's health through medical observation of newborns, and infant, reduction of child mortality, provision of adequate prenatal and postpartum health care services, and provision of information to parents regarding breastfeeding, hygiene and sanitation (7).

Fortification of Baking Flour – Law (2009 – present)
This law ensures flour fortification with Vitamins B_1, B_2, B_3, folic acid, and the minerals iron and zinc, with the goal of reducing micronutrient deficiency burden. Technical regulation was introduced in 2012 which regulated packing and labelling requirements, safety requirements, and storage and transportation guidelines (8,9).

Healthy Children at the Level of Primary Health Care (Regulation 589) (2010 – present)
First issued in 2010 and reformed in 2015, this order includes the classification of childhood periods, child health monitoring from 0-17 years of age, assessment of the development of children 0-6 years of age and instructions on breastfeeding and complementary feeding until two years of age. The main goal of this order is the assessment of a child's nutritional status as a preventative measure to ensure good health (10).

ii. Policies

National Reproductive Health Strategy (2006-2015)
Kyrgyzstan's National Reproductive Health Strategy has the objective of ensuring reproductive rights, and improvement of reproductive health for the Kyrgyz Republic. The priorities for this strategy include safe motherhood, reproductive choice, adolescent reproductive health, reproductive system cancers, prevention of violence, and the fight against STIs and HIV (11). Oblast Coordinating Councils of Reproductive Health were created as a means to address issues related to health organizations,

including raising awareness on sexual reproductive health, HIV, STIs and contraception (12). The primary focus of this strategy is to ensure human rights, gender equity and equity of service for socially vulnerable groups (13).

"Healthy Nation" State Plan (1994 – 1999)
The Healthy Nation State Plan was the first health policy of Kyrgyzstan and led to the implementation of a comprehensive health reform. The healthcare system was downscaled from previous soviet levels as this proved unaffordable for the newly independent Kyrgyz state. The goal of this plan was to build a foundation upon which the Kyrgyz people can build healthy lifestyles through physical improvement and spiritual development. The aims included the reduction of tuberculosis, disabilities, under-5 mortality, and maternal mortality. This plan set the stage for the reformation of the primary health care system, and the ultimate establishment of Manas (14,15).

National health reform programs "Manas" (1996 – 2006)
Manas reformed the health care delivery system with the aim of strengthening the primary health care system, developing family medicine and restructuring the hospital sector. This program improved the provision of pharmaceuticals, the quality of programs for maternal and child health, cardiovascular disease, TB, and HIV/AIDS. Health financing was refinanced and outcome based payment methods were introduced. *Manas* has since become an internationally recognized health financing model (16–20).

National Poverty Reduction Plan "Araket" (1998 – 2005)
This plan was the first attempt the Kyrgyz government made to reduce poverty in a comprehensive way. Araket created employment opportunities, and social and labor protection. This resulted in increased employment in rural areas through agrarian land reform, water and sewage costs were reduced, benefits for unemployment were provided, and there was an increase in the average size of pensions (21,22).

National health reform program "Manas Taalimi" (2006 – 2010)
Manas was reformed into *Manas Taalimi*, which implemented a sector wide approach (SWAp). Like its predecessor, *Manas Taalimi* aimed to improve the quality of health services at the primary health care level, to strengthen public health, and to improve the quality of medical education. Additional objectives of *Manas Taalimi* included the improvement of rural health centres, solidification of health financing reforms, and improvements in specialized care (16–19)**.**

National health reform program "Den Sooluk" (2012- 2016)
The *Den Sooluk* health reform program is based on three interrelated pillars: (1) expected health gain, (2) improving core services needed to achieve expected health gains, and (3) removing barriers that prevent the delivery of core services and health improvements. It aims to improve health outcomes of common diseases included maternal and child health, cardiovascular disease, TB, and HIV. *Den Sooluk* is based off both the success and shortcomings of *Manas* and *Manas Taalimi*, and it places a focus on improving social determinants of health (17,23–25).

This program aims to decrease maternal mortality through key population interventions including improvement of awareness regarding anemia, maternal nutrition, pregnancy danger signs, and health-seeking behaviours. It also provides individual services to achieve this goal, which include antenatal care, family planning services, active management of the third stage of labor to reduce postpartum haemorrhage, and high-quality emergency obstetric care. The *Den Sooluk* program recognizes that continuity of care for women during pregnancy, childbirth and postpartum is necessary, and includes a focus on improving interactions between different levels of care, as well as pilot testing new modes of service delivery (25).

Comprehensive development framework (CDF) of the Kyrgyz Republic (2002 – 2010)
The comprehensive development framework (CDF) is a World Bank initiative that was part of a long-term development strategy focusing on social and economic development. The CDF had the goal of poverty reduction by 50% by 2010. The CDF also focuses on the promotion of a healthy lifestyle, improvement in safe drinking water access, and strengthening the capacity of health and medical workers. Additionally, this framework focused on the community-based early childhood development project, child health and nutrition, supporting early childcare and education through kindergartens and pre-schools (26–28). The CDF ran between 2002 – 2010, but its implementation was interrupted after the tulip revolution and change in government in 2005.

National "A Promise Renewed" Initiative (2012 – present)

The “A Promise Renewed” initiative was borne out of the Child Survival Call to Action. Kyrgyzstan is one of over 30 countries that have committed to improving child survival based upon core principles including: (1) fostering political commitment to ending preventable child mortality through ambitious, measurable goals; (2) improving public accountability by monitoring and tracking activities that identify priorities for action; and (3) mobilizing communities to take action on improving child survival while holding governments accountable (29).

iii. Programs

WHO/UNICEF Breastfeeding Advocacy Program (1996-present)
UNICEF has long supported breastfeeding, and has made strides in promoting breastfeeding since the Innocenti Declaration on the Protection, Promotion and Support of Breastfeeding in 1990 (30). The Kyrgyz Order N19 on the Protection of Breastfeeding was the result of the UNICEF/WHO advocacy campaign.

Conditional Cash Transfer Program: Universal Monthly Benefit (UMB) (1995 – present)
The Universal Monthly Benefit (UMB) was introduced in 1995 with the purpose of providing aid that targeted children in impoverished families (2). The number of children a family has is proven to correlate significantly with poverty, which justifies the UMB’s focus on children. Children aged 1.5 to 16 years old were targeted, and if the child remained a student, the safety net was extended until age 21.

Conditional Cash Transfer Program: Monthly Social Benefit (MSB) (1998 – present)
The Monthly Social Benefit (MSB) was established in 1998 and served as a cash income replacement program targeting disadvantaged groups which include children with disabilities up to 18 years of age, other people with disabilities, orphaned children, mothers of large families, and elderly people who don’t qualify for pensions (2).

Mandatory Health Insurance Fund (MHIF) (1997 – present)
The Mandatory Health Insurance Fund (MHIF) program aimed to pool public funds in order to increase health system resources. The MHIF provided funding and provision of the essential package of health services (SGBP). Funding at local health facilities for drugs, medical supplies was improved by copayments, and aimed to decrease informal, out-of-pocket payments among individuals. The MHIF was a key component of the broader *Manas* health sector reform.
Primary Health Care Reforms & Introduction of Family Medicine Model (1997 – present)
This model reformed the centrally managed specialist-based primary care in place during Soviet rule into an approach that employs a comprehensive family medicine model (31). Mid-level general practitioners were trained to provide primary health care at the community level, which was a substantial gap in the post-Soviet health system. In addition, family medical centres and family group practices were established (31). Kyrgyzstan has since been recognized as a regional leader in family medicine reform (32).

State Guaranteed Benefits Package & Additional Drug Package (2000 – present)
The State Guaranteed Benefits Package (SGBP) defined an essential package of health services such as primary health care, and emergency care to be provided to all Kyrgyz citizens free of charge. Secondary care was subsidized and official copayments were introduced, with substantial exemptions for vulnerable populations. The Additional Drug Package (ADP) was also introduced and it partially subsidized drug costs using the MHIF increasing drug accessibility and quality to the Kyrgyz people. Medicines included on the ADP do not fully align with medicines included in WHO’s Model Lists of Essential Medicines (33). These programs were funded through the MHIF and helped to decrease informal and out-of-pocket payments, and increased accessibility and utilization of primary care services among the population. The SGBP is seen as the critical step/strategy of *Manas* health sector reforms and has contributed towards sustaining universal health coverage.

National Perinatal Care Program/Strategy (2008-present)
The national perinatal care program is intended to reduce maternal, perinatal, neonatal and infant mortality by increasing the quality of medical care provided to mothers and newborns and ensuring equal access in all regions of the country. This falls under the Den Sooluk Health Reform Programme, which has an objective to improve obstetric care through provision of individual services and access to quality health services at all levels of an integrated perinatal care system. The National Perinatal Care Strategy has 5 objectives:

- Objective 1: Building a multi-level system of perinatal/neonatal care
- Objective 2: Establishment of transportation/counselling system
- Objective 3: Perinatal care quality improvement through improvement of professional knowledge and practical skills of the health professionals
- Objective 4: Development of monitoring and evaluation system (audit) for perinatal/neonatal care quality
- Objective 5: Establishment of a differentiated payment system for perinatal care based on different packages of services, depending on case risk or severity (34)

The UNICEF Delivering as One (DaO) and Equity projects assist the National Perinatal Care Strategy in achieving Objectives 1, 3 and 4 (35). This strategy supported three main training courses: Effective Perinatal Care (EPC), Neonatal Resuscitation (NR), and Effective Neonatal Care (ENC) (34).

Village Health Committees & Community Action for Health Model/Program (2001 – present)
The Community Action for Health Model introduced community health workers in independent community-based organizations called Village Health Committees. The community health worker volunteers are trained by health providers and contribute to disease prevention and health promotion in communities, with a particular focus on exclusive breastfeeding. This programme aimed to improve population and community involvement (a key component of the Manas Taalimi reform) and is recognized by the Ministry of Health as the main strategy for community mobilization and health promotion by the Ministry of Health (16). UNICEF linked with VHCs in order to support nutrition communication activities and deliver quality education and counseling on issues related to nutrition (2).

PMTCT Programme (2012-present)
HIV/AIDS is one of four program areas that were selected by Den Sooluk as priority areas to achieve better overall health outcomes (25). As part of this programme, the prevention of mother-to-child transmission (PMTCT) is highlighted, with an aim to target HIV testing toward high risk pregnant women, and wide implementation of early infant diagnosis. Antenatal HIV testing is free in Kyrgyzstan, and nearly 88% of pregnant women are tested, with rapid tests available at maternal houses. Kyrgyzstan's PMTCT programme has led to few vertical transmissions since 2012, though approximately 5% of children born to pregnant women living with HIV become infected (36).

Every Newborn Action Plan (2014-present)
In 2014, the WHO member states endorsed the Every Newborn Action Plan (ENAP) which provides strategic actions to end preventable newborn mortality and stillbirths, as well as to reduce maternal mortality and morbidity. This plan provides eight specific milestones for the improvement of maternal and newborn health by 2030. Thus far, Kyrgyzstan has developed a national newborn action plan, adopted a policy for maternal death notification, developed skilled birth attendant retention and training policies, and has an MNH engagement/mobilization strategy. However, Kyrgyzstan has not yet developed a national communication strategy on newborn development, nor put in place a perinatal death system review (37).

Perinatal Audit (2018-present)
In 2016, the perinatal audit tool was launched in Uzbekistan. Representatives from Kyrgyzstan were present at the meeting based upon a prior request for WHO assistance in this area (38). This tool was developed to help with using a perinatal audit to improve maternal and newborn health care, and is outlined in the WHO publication “Making every baby count: audit and review of stillbirths and neonatal deaths”. Piloting of this programme began in Kyrgyzstan in 2018 with the help of UNICEF, under the direction of the MoH. This initiative will help to identify barriers and gaps in services, and will also add qualitative data related to perinatal deaths.

Baby Friendly Hospital Initiative (2000– present)
The Baby Friendly Hospital Initiative aimed to implement the 10 principles of successful breastfeeding in maternal hospitals, and to regulate the organization of a dairy room for the storage of pasteurized milk in maternity hospitals (5). In 2009, this initiative was amended to include an 11th step prohibiting the use of breastmilk substitutes.

Program on prevention of iron deficiency anemia among the population in Kyrgyzstan (1995 – 2016)

This program was designed to prevent anemia through flour fortification, preventative supplements for at risk groups, dietary diversity promotion, minimizing and controlling infections, the dissemination of monitoring and research, and linking anemia control to public health services (24).

"Jan-ene" program (2002 – 2006)

The *Jan-ene* program has a focus on the promotion of perinatal care, gender equality, and the improvement of the genetic pool. It aims to improve the reproductive health of girls, and women, and to reduce infant and maternal mortality. Improvements were also made in the training and professional development of personnel and provide public awareness on safe motherhood and women's health (39).

The National Poverty Reduction Strategy (NPRS) (2003 – 2005)

The first step to implementing the Comprehensive Development Framework was the National Poverty Reduction Strategy (NPRS). The NPRS centres on enhancing economic growth, adding employment opportunities, improving efficiency and targeting of social protection and human development, and improving institution and human capacity. As NPRS was implemented, reforms were carried out, which ensured macroeconomic stability, and resulted in average annual economic growth of 5%. Kyrgyz people's incomes increased, health and education services improved, which led to a poverty reduction from 52% to 44% (21,22,40).

Integrated management of childhood illness program (2005 – present)

The IMCI program targets medical school students and post-graduates with the aim of improving the medical supervision of sick children in order to reduce child and infant mortality. This program gave medical professionals greater confidence in treating children under 5, which led to devotion of more time directly to the child while filling in their records; parents beginning to bring their children in for treatment earlier in their illness and thus presenting less seriously ill children; parents asking for children to be weighed; fewer unnecessary medicines being used; and mothers receiving more advice on how to nurse a sick child and care for a healthy one. As a result, mothers liked the new ways in which children were received, more mothers were breastfeeding long term, and more children were receiving only breast-milk, while fewer children were being referred for hospitalization (41).

Gulazyk supplementation (2009 – present)

Gulazyk is a program of home fortification meant to deliver micronutrients to children between 6-24 months of age. The English translation is Gulazyk is a meat product rich in energy and nutrients. The Ministry of Health initiated this program with the help of UNICEF in response to high anemia levels in young children. Gulazyk is a powder that contains iron, zinc, Vitamin A and C and was distributed by trained primary care providers at family health clinics (42–44).

Quality Health Care Project (QHCP) (2010 – 2015)

The QHCP included five main components that were considered the most pressing health issues Kyrgyzstan faced at this time, which included TB prevention and treatment, HIV prevention and access to care, improvements to maternal and child health, increased used of family planning and reproductive health services, and addressing anti-microbial resistance. The most successful component was the TB component (45–50). The QHCP supported the WHO program on effective perinatal care, which impacted maternal and child health through all maternity and delivery facilities in Kyrgyzstan. The QHCP helped improve hospital care of infants and children through support of the WHO program on management of childhood illnesses. QHCP also delivered training on emergency management of obstetric complications to medical staff (48).

Table 6: Categorization of laws, policies and programs in Kyrgyzstan as related to the seven LiST packages

PR	Poverty Reduction: laws, policies, and programs that reduce poverty
HSS	Health Systems Strengthening: laws, policies, and programs that broadly strengthen the health system
CH	Child Health: laws, policies, and programs that are related to child health, but do not specify essential newborn health package interventions
MH	Maternal Health: laws, policies, and programs that are related to maternal health

	Preconception nutrition care	Antenatal care	Advanced antenatal care	Care during labour and child birth	Immediate newborn care	Care of the normal newborn	Care of the small and sick newborn
Laws, legislations, decrees							
State Benefits law (1991-present) [PR]							
Order N 19: Protection of Breastfeeding (1996 – present)							
Protection of Breastfeeding of Children and the Regulation of Marketing of Infant Food (2008 – present)							
Code of Kyrgyz Republic of children (2006 – present) [CH]							
Fortification of Baking Flour – Law (2009 – present)							
Healthy Children at the Level of Primary Health Care (Regulation 589) (2010 – present) [HSS]							
Policies							
National Reproductive Health Strategy (2006-2015) [MH]							
"Healthy Nation" State Plan (1994 – 1999) [HSS]							
National health reform programs "Manas" (1996 – 2006) [HSS]							
"A Promise Renewed" Initiative (2012-present)[CH]							
Poverty reduction: Araket (1998-2005) [PR]							
Manas Taalimi (2006 – 2010) [HSS]							

	Preconception nutrition care	Antenatal care	Advanced antenatal care	Care during labour and child birth	Immediate newborn care	Care of the normal newborn	Care of the small and sick newborn
National health reform program "Den Sooluk" (2012 – present)[HSS]							
Comprehensive development framework (CDF) of the Kyrgyz Republic (2002 – 2010) [PR]							
Programs							
WHO/UNICEF Breastfeeding Advocacy Program							
Conditional Cash Transfer Program: Universal Monthly Benefit (1995-present) [PR]							
Conditional Cash Transfer Program: Monthly Social Benefit (1998-present) [PR]							
Mandatory Health Insurance Fund (MHIF) (1997 – present) [HSS]							
Primary Health Care Reforms & Introduction of Family Medicine Model (1997 – present) [HSS]							
State Guaranteed Benefits Package & Additional Drug Package (2000 – present) [HSS]							
National Perinatal Care Program/Strategy (2008-present) [HSS]							
Village Health Committees & Community Action for Health Model/Program (2001 – present) [CH, MH]							
PMTCT Programme (2012-present)[CH]							
Every Newborn Action Plan (2014-present)[CH]							
Perinatal Audit (2018-present)[CH]							
Baby Friendly Hospital Initiative (2000–present)							
Program on prevention of iron deficiency anemia among the population in Kyrgyzstan (1995 – 2016)							
"Jan-ene" program (2002 – 2006) [MH]							

	Preconception nutrition care	Antenatal care	Advanced antenatal care	Care during labour and child birth	Immediate newborn care	Care of the normal newborn	Care of the small and sick newborn
Integrated management of childhood illness program (2005 – present)							
Gulazyk supplementation (2009 – present) [CH]							
Quality Health Care Project (QHCP) (2010 – 2015) [HSS]							
National Poverty Reduction Strategy (2003-2005) [PR]							

Figure 25: ODA received and disbursed on RMNCH

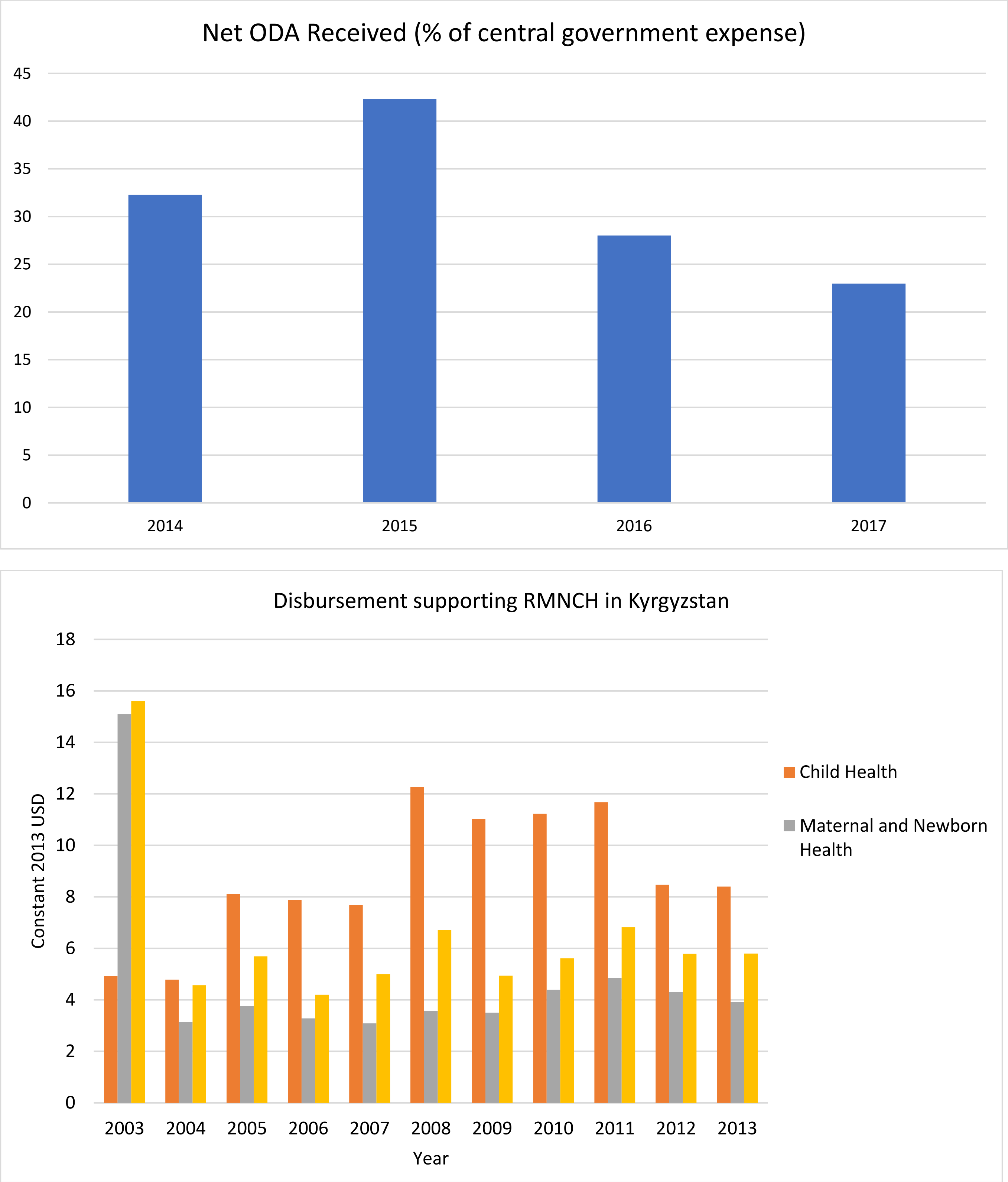

Table 7: Intervention coverage assumptions for LiST analysis

Intervention (%)	Coverage at baseline	2025 coverage target	2030 coverage target	Source
**PRECONCEPTION & NUTRITION CARE**				
Folic acid supplementation/fortification	0.0	90.0	99.0	LiST default
Blanket iron supplementation/fortification	0.0	90.0	99.0	LiST default
Calcium supplementation	0.0	90.0	99.0	LiST default
Micronutrient supplementation (iron and multiple micronutrients)	2.3	90.0	99.0	LiST default
Balanced energy supplementation	0.0	90.0	99.0	MICS 2018
**ANTENATAL CARE**				
TT - Tetanus toxoid vaccination	0.0	90.0	99.0	MICS 2018
Syphilis detection and treatment	8.2	90.0	99.0	LiST default
**ADVANCED ANTENATAL CARE**				
Hypertensive disorder case management	70.6	90.0	99.0	LiST default
Diabetes case management	35.3	90.0	99.0	LiST default
Malaria case management	78.2	90.0	99.0	LiST default
MgSO4 management of pre-eclampsia	70.8	90.0	99.0	LiST default
Fetal growth restriction detection and management	0.0	90.0	99.0	LiST default
**CARE DURING LABOUR & CHILD BIRTH**				
Skilled birth attendance	99.7	NA	NA	MICS 2018
Health facility delivery	99.6	NA	NA	MICS 2018
**CARE OF THE NORMAL NEWBORN**				
Early initiation of breastfeeding	83.6	90.0	99.0	MICS 2018
**Breastfeeding(< 1 month)**				
Exclusive breastfeeding	57.4	90.0	99.0	LiST default
Predominant breastfeeding	26.2	90.0	99.0	LiST default
Partial breastfeeding	12.0	90.0	99.0	LiST default
Not breastfeeding	4.4	90.0	99.0	LiST default
Clean postnatal practices	79.8	90.0	99.0	LiST default
Chlorhexidine	0.0	90.0	99.0	LiST default
**CARE OF SMALL & SICK NEWBORN**				
Case management of premature babies (KMC, thermal care)	0.0	90.0	99.0	LiST default
Case management of neonatal sepsis/pneumonia (Oral/injectable antibiotics, full supportive care)	0.0	90.0	99.0	LiST default
ORS - oral rehydration solution	35.2	90.0	99.0	MICS 2018

Table 8: Lives saved by Every Newborn Action Plan (ENAP) interventions

	2018-2025		2018-2030	
	Lives Saved	%	Lives Saved	%
Neonatal	584	36.4	624	38.9
Stillbirth	151	9.8	172	11.2

Maternal	19	16.8	21	18.6

Lives saved by interventions						
	Neonatal		Stillbirth		Maternal	
	2025	2030	2025	2030	2025	2030
**Periconceptual**						
Folic acid supplementation/fortification	42	45	0	0	0	0
**Pregnancy**						
TT - Tetanus toxoid vaccination	3	3	0	0	0	0
Syphilis detection and treatment	0	0	1	1	0	0
Calcium supplementation	0	0	0	0	4	4
Micronutrient supplementation (iron and multiple micronutrients)	28	29	92	95	7	7
Balanced energy supplementation	5	6	22	27	0	0
Diabetes case management	0	0	12	13	0	0
MgSO4 management of pre-eclampsia	0	0	24	35	4	6
Hypertensive disorder case management	0	0	0	0	3	4
**Breastfeeding**						
Age-appropriate breastfeeding practices	15	16	0	0	0	0
**Preventive**						
Clean postnatal practices	10	17	0	0	0	0
Chlorhexidine	34	35	0	0	0	0
**Curative after birth**						
Case management of premature babies	298	321	0	0	0	0
Case management of neonatal sepsis/pneumonia	148	150	0	0	0	0
ORS - oral rehydration solution	2	2	0	0	0	0

Figure 26a: Total maternal lives saved by intervention in 2018-30, at the national level

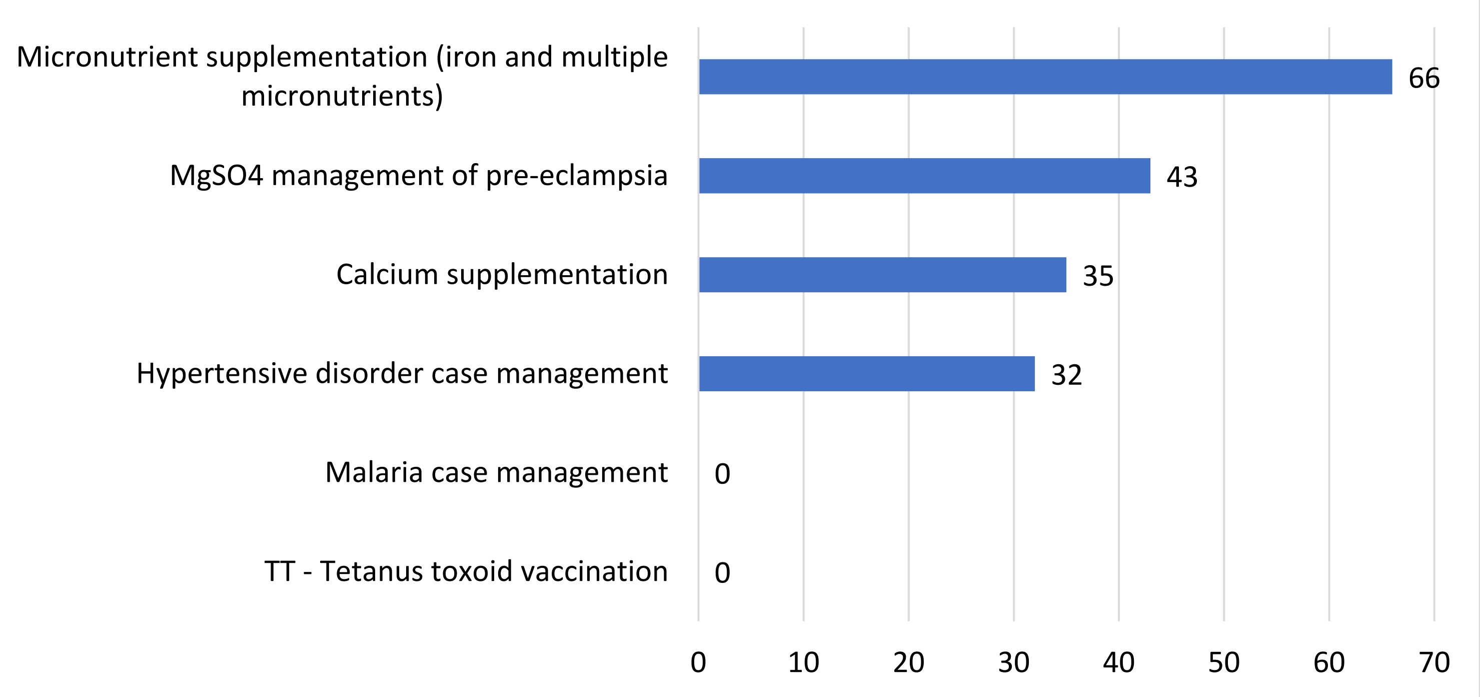

Figure 26b: Maternal lives saved annually by intervention in Kyrgyzstan, 2018-2030

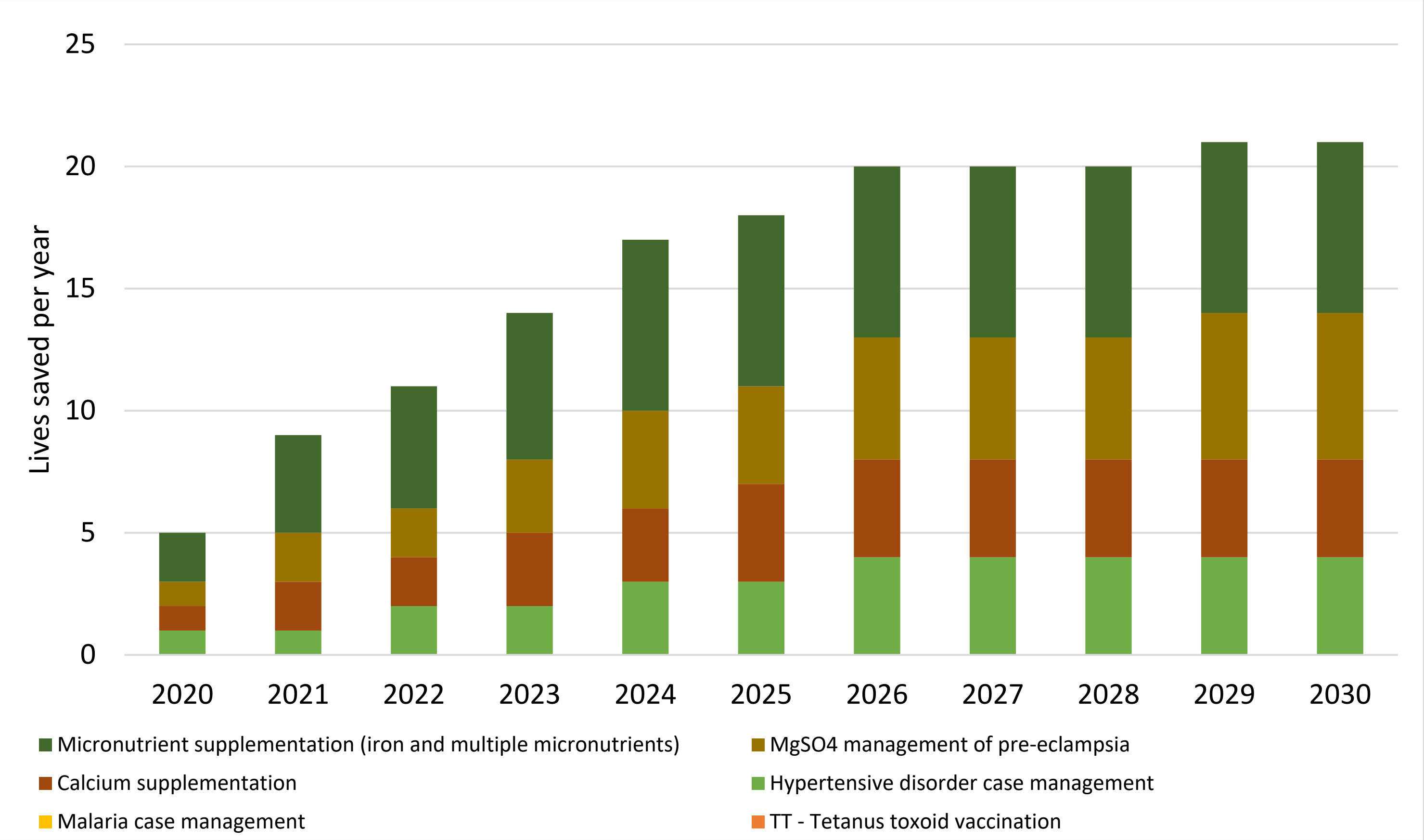

Figure 27a: Total stillbirths saved by intervention in 2018-30, at the national level

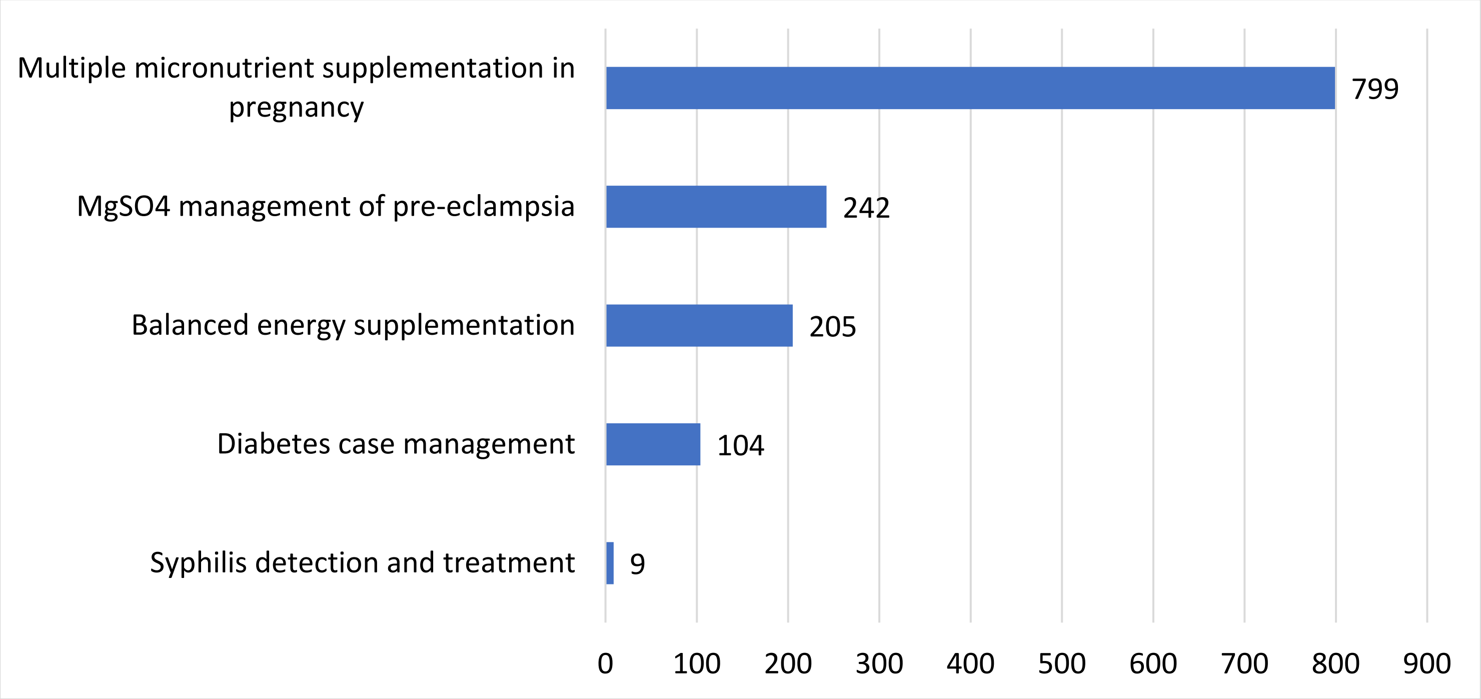

Figure 27b: Stillbirths saved annually by intervention in Kyrgyzstan, 2018-2030

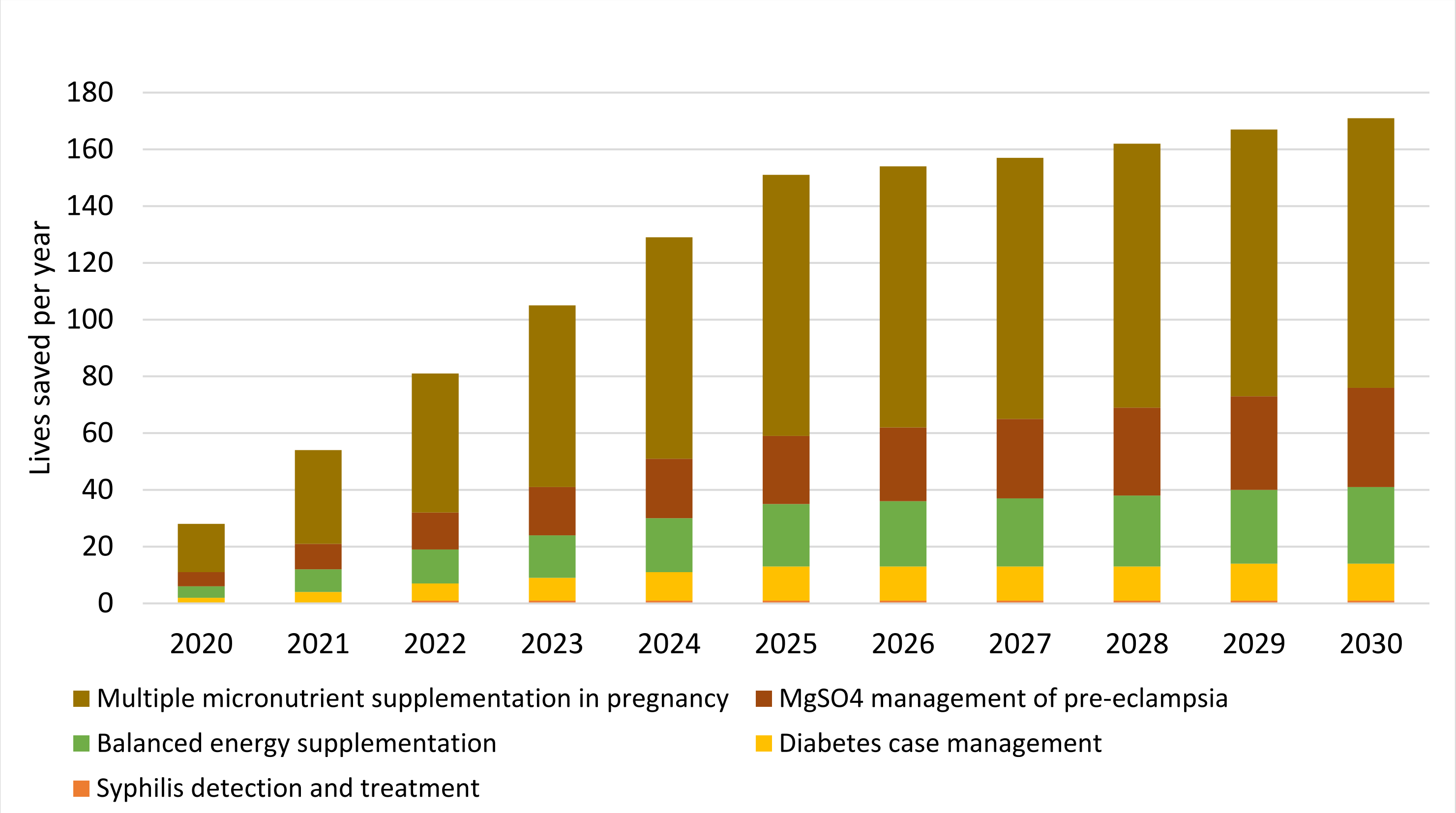

Table 9: Documents and Reports Reviewed

Author/Institution	Title	Year
A.Liubsys/UNICEF	A Model of Multi-level System of Perinatal/Neonatal Health Care Delivery in the Kyrgyz Republic	2008
S. Newport et al./UNFPA	A Review of Progress in Maternal Health in Eastern Europe and Central Asia	2009
I. Lejnev & A. Kuttumuratova/WHO	Approaches to an integrated supervisory system in Kyrgyzstan for better maternal and child health	2009
W. Lutz/UNFPA	Emerging Population Issues in Eastern Europe and Central Asia	2010
Asian Development Bank	Country Operations Business Plan – Kyrgyz Republic 2011-2012	2011
A. Ibraimova et al./WHO	Kyrgyzstan Health System Review. Health Systems in Transition	2011
T. Rabie et al./World Bank	Situational Analysis Improving economic outcomes by expanding nutrition programming in the Kyrgyz Republic	2011
Asian Development Bank	Management Response to the country assistance program evaluation for Kyrgyz Republic	2012
J. Cekota et al./UNDP	The United Nations Economic Commission for Europe (UNECE) Report on Achieving the Millennium Development Goals in Europe and Central Asia, 2012	2012
T. Wardlaw et al./UNICEF	Committing to Child Survival: A promise renewed – progress report 2012	2012
Alberta Bacci et al./MOH, WHO, UNFPA, UNICEF	Improving quality of care for mothers and newborn babies in the Republic of Kyrgyzstan Assessment of quality of maternal and neonatal services at hospital and primary health care levels	2012
UNICEF	Cost benefit analysis of the equity programme in southern Kyrgyzstan- Centre for Socioeconomic Research	2012
DFID/Coffey International Development	Public Expenditure Tracking Survey (PETS) on Health and Education in Kyrgyz Republic- Health Service Delivery Report	2012
R. Hasanov et al./UNDP	The Kyrgyz Republic Third Report on Progress towards achieving the millennium development goals 2013	2013
World Bank	Kyrgyz Republic Early Childhood Development SABER Country Report 2013	2013
Curatio International Foundation	Progress in Reducing Health System Bottlenecks Towards Achieving the MDG4: Evaluation of UNICEF's Contribution in Five CEE/CIS Countries	2013
MoH/MoE	Kyrgyzstan MDG Acceleration Framework	2013
Norwegian Helsinki Committee & UNFPA	Analysis of Early Marriage and Early Maternity in Kyrgyzstan	2013
Gulgun Murzalieva et al./ Health Policy Analysis Center	Research note on Maternal Mortality in the Kyrgyz Republic	2013
UNICEF	Regional Analysis Report 2013 (Regional Office for Central and Eastern Europe and the Commonwealth of Independent States (CEE/CIS))	2014
T. Gotsadze & C. Zanetti/UNICEF	Evaluation of UNICEF programme on perinatal care for the period 2010-2013 project evaluation report	2014

Author/Institution	Title	Year
Republican Centre for Health Promotion (RCHP)	Assessment of knowledge and awareness of danger signs of pregnancy and childhood diseases in communities of Osh, Batken, and Jalal-Abad provinces	2014
T.Gotsadze/UNICEF	Evaluation of UNICEF Programme on Perinatal Care for the period 2010 – 2013	2014
WB Kyrgyzstan	Kyrgyz Republic: Performance Based payments for maternal and neonatal health- Quantitative baseline survey report	2014
J. Azevado et al./World Bank	Kyrgyz Republic Social Sectors at a Glance	2015
Tim Cammack et al./ UNICEF	Strengthening equity focused budgeting for Maternal, Newborn and Child Health (MNCH) in Central Asia	2015
T. Gotsadze & K. Shalyeva/UNICEF	Evaluation of UNICEF Country Program (2012-2016) and Strategic Positioning	2016
P. Schneider & S. Vogler/WHO	Pharmaceutical pricing and reimbursement reform in Kyrgyzstan	2016
G. Najimidinova/UNICEF	Analytical Review of Documents on the System of Home Visits in the Kyrgyz Republic	2016
Asian Development Bank	Asian Development Bank Member Fact Sheet – Kyrgyz Republic	2017
UNICEF	Country Programme document (2018-2022) – Kyrgyzstan	2017
UNICEF	Regional Analysis Report 2016 (Regional Office for Europe and Central Asia (CEE/CIS)	2017
UNICEF	UNICEF Annual Report 2017 – Kyrgyzstan	2017
D. Collins et al./WHO	Improving the implementation of a package of essential non-communicable (PEN) disease interventions in Kyrgyzstan: mixed methods service evaluation of pilot health centres	2017
D. Nassler, K. Hornetz/GIZ	Strategic Outline for the Capacity Development Plan for the National Perinatal Care Centre (NPC)	2017
T. Gotsadze, G. Murzalieva/Swiss Embassy	Impact evaluation of the Community Action for Health (CAH) project in Kyrgyzstan	2017
D. Nassler, K. Hornetz/GIZ	Capacity Development Planning Support to the Bishkek Maternity Hospital of the National MCH Center (NMCHC)	2017
UNICEF, WHO, IPA, MOH	Inter-country Meeting to Accelerate progress in MNH in Central Asia and Caucasus in the context of the SDGs Bishkek, Kyrgyzstan -- Meeting Report	2017
C. Aidyralieva/GIZ	Analysis of the data and documents on the perinatal care in Kyrgyzstan	2017
G. Siupsinskas/GIZ	Promotion of Perinatal health in Kyrgyzstan	2017
Asian Development Bank	Country Partnership Strategy Kyrgyz Republic, 2018–2022 —Supporting Sustainable Growth, Inclusion, and Regional Cooperation	2018
UNICEF	ROAR 2017: Europe and Central Asia Regional Office Annual Report	2018
WHO	A scoping review on health services delivery in Kyrgyzstan: what does the evidence tell us?	2018
C. Shaw et al./WHO	Quality of care review in Kyrgyzstan	2018
WHO	The World Health Organization in Kyrgyzstan	2018
MoH	Every Newborn Action Plan (ENAP) Simplified Country Implementation Tracking Tool	2018
T. Gotsadze /UNICEF	Introduction of 24 hour pediatric hospitalization practice in Republic of Kyrgyzstan	2018
GIZ/EPOS Health/ in coordination with CAI	Promotion of Perinatal Health in Kyrgyzstan - Perinatal Care Referral System Study	2018

References

1. Victora C G, Huttly, S R, Fuchs, S C, Olinto, M T. The role of conceptual frameworks in epidemiological analysis: a hierarchical approach. Int J Epidemiol. 1997;26(1):224–7.
2. World Bank, UNICEF. Situational Analysis: Improving economic outcomes by expanding nutrition programming in the Kyrgyz Republic. Washington, D.C.; 2011.
3. World Food Programme. Emergency Food Security Assessment (EFSA). 2012.
4. Dhur A. Food Security Assessment in the Kyrgyzstan Republic. 2008.
5. UNICEF, WHO. Country experiences with the baby-friendly hospital initative [Internet]. New York, New York; 2017. Available from: https://www.unicef.org/nutrition/files/BFHI_Case_Studies_FINAL.pdf
6. Justice M of. Law on protection of breastfeeding and regulation of marketing of infant food. Kyrgyzstan: Ministry of Justice; 2008.
7. Code of the Kyrgyz Republic of Children № 151 [Internet]. Bishkek: Ministry of Justice of the Kyrgyz Republic; 2006 [date accessed 2019 Mar 28]. Available from: http://cbd.minjust.gov.kg/act/view/ru-ru/1949?cl=ru-ru#7
8. Law on the Fortification of Baking Flour. Kyrgyzstan; 2009.
9. Shevchuk S, Ghauri K. Afghanistan/Central Asia Regional Food Fortification Program [Internet]. Geneva, Switzerland; 2015. Available from: https://www.gainhealth.org/wp-content/uploads/2014/07/Analysis-of-Food-Fortification-in-CAR-Afghanistan-and-Pakistan-ENG.pdf
10. Kyrgyz Republic. Law on state benefits for families with children. Bishkek; 1991.
11. National Strategy for the Protection of Reproductive Health of the Population of the Kyrgyz Republic until 2015 (July 15, 2006 No. 387) [Internet]. Ministry of Justice of the Kyrgyz Republic; 2006 [date accessed 2019 Mar 28]. Available from: http://cbd.minjust.gov.kg/act/view/ru-ru/4506?ckwds=%2B%25d0%25bd%25d0%25be%25d0%25b2%25d0%25be%25d1%2580%25d0%25be%25d0%25b6%25d0%25b4%25d0%25b5%25d0%25bd%25d0%25bd%25d1%258b%25d0%25b9
12. Hasanov R, Choibaeva N, Dyikanbaeva G, et al. Third Report on Progress Towards Achieving the Millenium Development Goals- UNDP [Internet]. Bishkek, Kyrgyz Republic; 2013 [date accessed 2019 Apr 10]. Available from: https://www.kg.undp.org/content/kyrgyzstan/en/home/library/mdg/third-mdg-progress-report-kyrgyzstan.html
13. UNFPA. A Review of Progress in Maternal Health in Eastern Europe and Central Asia [Internet]. 2009 [date accessed 2019 Apr 15]. Available from: https://www.unfpa.org/publications/review-progress-maternal-health-eastern-europe-and-central-asia
14. Huseynov S, Steinglass R. Immunization and health sector reform in the Kyrgyz Republic [Internet]. World Health Organization. Geneva, Switzerland; 1999. Available from: http://unpan1.un.org/intradoc/groups/public/documents/apcity/unpan046798.pdf
15. Ministry of Justice of the Kyrgyz Republic. Government decree state program “Healthy Nation 1994-2000.” Bishkek;
16. Ibraimova A, Akkazieva B, Ibraimov A, Manzhieva E, Rechel B. Kyrgyzstan: Health system review [Internet]. Vol. 13, Health systems in transition. 2011 [date accessed 2019 Apr 14]. p. xiii, xv–xx, 1–152. Available from: https://www.euro.who.int/__data/assets/pdf_file/0017/142613/e95045.pdf
17. WHO. Den Sooluk National Health Reform Program in the Kyrgyz Republic for 2012-2016 [Internet]. 2011 [date accessed 2019 Apr 16]. p. 1–59. Available from: https://extranet.who.int/nutrition/gina/sites/default/files/KGZ 2012-2016 Den Sooluk - National Health Plan.pdf
18. UNICEF. SITUATION ASSESSMENT OF CHILDREN IN THE KYRGYZ REPUBLIC. 2011;255.
19. UNICEF. Maternal and Newborn Health in Chui Province & Kyrgyzstan: Assessment and Implications for Interventions. Bishkek, Kyrgyz Republic; 2009.
20. Saikal M. Pros and cons of national health reform programs “Manas”, “Manas Taalimi” and “Den sooluk.” Bishkek;
21. World Bank. Participatory Poverty Assessment in the Kyrgyz Republic For the World Development Report 2000/01

[Internet]. Bishkek, Kyrgyz Republic; 1999. Available from: http://siteresources.worldbank.org/INTPOVERTY/Resources/335642-1124115102975/1555199-1124138866347/kyrgyz.pdf
22. Yamaguchi A, Danilenko A. Water and Sewerage Utilities in the Kyrgyz Republic: Performance INdicators.
23. WHO/Kyrgyz Republic Health Policy Analysis Centre. Integration of the Sustainable Development Goals 2030 in the strategic programs of Kyrgyzstan ' s healthcare sector and the country ' s Development Strategy 2030. Bishkek, Kyrgyzstan; 2017.
24. Novovic T, Ibraeva G, Gabdulhakov R. Mid Term Review of the United Nations Development Assistance Framework (UNDAF) for Kyrgyz Republic (2012 - 2017). Bishkek, Kyrgyzstan; 2015.
25. World Health Organization. Den Sooluk National Health Reform Program in the Kyrgyz Republic for 2012 - 2016 [Internet]. Global database on the Implementation of Nutrition Action (GINA). 2012. Available from: https://extranet.who.int/nutrition/gina/en/node/23556
26. World Health Organization. Comprehensive Development Framework of the Kyrgyz Republic [Internet]. 2003. Available from: https://extranet.who.int/nutrition/gina/sites/default/files/KGZ 2002 Comprehensive Development the Framework of Kyrgyz Republic to 2010.pdf
27. Jacobs C. Evaluating the Comprehensive Development Framework in Kyrgyz Republic, Central Asia Magic Bullet or White Elephant? Evaluation [Internet]. 2005;11(4):480–95. Available from: http://www.kyrgyzinvest.kg
28. Kyrgyzstan Development Gateway. Monitoring and Evaluation [Internet]. Comprehensive Development Framework for the Kyrgyz Republic. 2001. Available from: http://eng.gateway.kg/content/strategies/cdf/92
29. Committing to Child Survival: A Promise Renewed [Internet]. [date accessed 2019 May 7]. Available from: www.apromiserenewed.org
30. UNICEF Innocenti Research Centre. Celebrating the Innocenti Declaration on the Protection, Promotion and Support of Breastfeeding Past Achievements, Present Challenges and the Way Forward for Infant and Young Child Feeding [Internet]. 1990 [date accessed 2019 Apr 3]. Available from: https://www.unicef.org/nutrition/files/Innocenti_plus15_BreastfeedingReport.pdf
31. Good Health at Low Cost': 25 years on. What makes a successful health system? [Internet]. Vol. 20, Reproductive Health Matters. 2012. 212–214 p. Available from: https://www.tandfonline.com/doi/full/10.1016/S0968-8080%2812%2939614-6
32. Hardison C, Fonken P, Chew T, Smith B. The emergence of family medicine in Kyrgyzstan. Fam Med. 2007;39(9):627–33.
33. Vogler S, Haasis MA, Dedet G, Lam J, Pedersen HB. Medicines Reimbursement Policies In Europe [Internet]. 2018. Available from: http://apps.who.int/medicinedocs/documents/s23486en/s23486en.pdf
34. Gotsadze T, Zanetti C. Evaluation of UNICEF programme on perinatal care for the period 2010-2013 project evaluation report [Internet]. 2014 [date accessed 2019 Apr 16]. Available from: https://www.unicef.org/evaldatabase/index_73583.html
35. Health Policy Analysis Centre. The quality of maternal and child health services provided in pilot hospitals in Osh, Jalalabad, Batken, and Issyk Kul oblasts (2014-2017) [Internet]. 2014. Available from: https://www.unicef.org/kyrgyzstan/sites/unicef.org.kyrgyzstan/files/2018-01/Q_REPORT EN.pdf
36. Mansfeld M, Ristola M. HIV Programme Review in Kyrgyzstan [Internet]. 2014 [date accessed 2019 May 7]. Available from: http://www.euro.who.int/pubrequest
37. World Health organization (WHO), UNICEF. Reaching Every Newborn National 2020 Milestones [Internet]. 2018 [date accessed 2019 May 7]. Available from: https://www.healthynewbornnetwork.org/hnn-content/uploads/Final-Country-Progress-Report-v9-low-res.pdf
38. New WHO perinatal audit tool in Russian is launched in Uzbekistan. 2016 Nov 9 [date accessed 2019 May 7]; Available from: http://www.euro.who.int/en/health-topics/Life-stages/maternal-and-newborn-health/news/news/2016/11/new-who-perinatal-audit-tool-in-russian-is-launched-in-uzbekistan
39. UNDP. The Kyrgyz Republic: The Second Progress Report on The Millennium Development Goals [Internet]. Bishkek, Kyrgyzstan; 2010. Available from: http://www.kg.undp.org/content/kyrgyzstan/en/home/library/mdg/the-first-millennium-development-goals-progress-report-in-the-ky.html
40. International Labor Organization, International Programme on the Elimination of Child Labour. Child Labour in Kyrgyzstan: An Initial Study. Bishkek; 2001.
41. World Health organization (WHO). Evaluation in Kyrgyzstan [Internet]. World Health Organization; 2018 [date accessed 2018 Jul 9]. Available from: http://www.euro.who.int/en/health-topics/Life-stages/child-and-adolescent-health/child-and-adolescent-health2/children-at-home-and-in-primary-health-care/evaluation-in-kyrguzstan

42. UNICEF. UNICEF Annual Report 2015 - Kyrgyzstan. 2015;1–29.
43. Lundeen E, Imanalieva C, Mamyrbaeva T, Timmer A. Integrating Micronutrient Powder into a Broader Child Health and Nutrition Program in Kyrgyzstan. In: De Pee S, Flores-Ayala R, Van Hees J, Jefferds ME, Irizarry L, Kraemer K, et al., editors. Home Fortification with Micronutrient Powders (MNP). Basel, Switzerland: Sight and Life, UNICEF, WFP, HF-TAG; 2013. p. 23–30.
44. Global Forum on Food Security and Nutrition in Europe and Central Asia. Food security and nutrition programme for Kyrgyzstan in action. How to implement policy in the most efficient way? 2016;1–6.
45. USAID. USAID Quality Health Care Project [Internet]. 2015. Available from: https://www.usaid.gov/kyrgyz-republic/fact-sheets/usaid-quality-health-care-project
46. USAID. Kyrgyz Ministry of Health supports expansion of successful USAID pilot for ambulatory treatment of TB [Internet]. Available from: https://www.usaid.gov/sites/default/files/documents/1861/Kyrgyz Ministry of Health supports expansion of successful USAID pilot for ambulatory treatment of TB.pdf
47. USAID, SPRING. Strengthening Nutrition within the Kyrgyz Republic Health System: SPRING in the Kyrgyz Republic (September 2017). Washington, D.C.; 2017.
48. U.S. Embassy in the Kyrgyz Republic. USAID presents results of Quality Health Care Project [Internet]. 2015. Available from: https://kg.usembassy.gov/usaid-presents-results-quality-health-care-project/
49. USAID. USAID improves quality of TB laboratory services [Internet]. Kyrgyz Republic. Available from: https://www.usaid.gov/sites/default/files/documents/1861/USAID improves quality of TB laboratory services.pdf
50. USAID. Patients help each other complete TB treatment [Internet]. Kyrgyz Republic. Available from: https://www.usaid.gov/sites/default/files/documents/1861/Patients help each other complete TB treatment.pdf
